# Automation of structural health monitoring (SHM) system of a bridge using BIMification approach and BIM-based finite element model development

**DOI:** 10.1038/s41598-023-40355-7

**Published:** 2023-08-14

**Authors:** Muhammad Fawad, Marek Salamak, Grzegorz Poprawa, Kalman Koris, Marcin Jasinski, Piotr Lazinski, Dawid Piotrowski, Muhammad Hasnain, Michael Gerges

**Affiliations:** 1 Faculty of Civil Engineering, Silesian University of Technology, Ul. Akademicka 2A, 44-100 Gliwice, Poland; 2 Faculty of Civil Engineering, Budapest University of Technology and Economics, Műegyetem Rkp. 3, 1111 Budapest, Hungary; 3 University of Florida, Gainesville, FL 32611 USA; 4 University of Wolverhampton, Wulfruna St, Wolverhampton, WV1 1LY UK

**Keywords:** Civil engineering, Electrical and electronic engineering

## Abstract

This research focuses on the automation of an existing structural health monitoring system of a bridge using the BIMification approach. This process starts with the Finite Element Analysis (FEA) of an existing bridge for the numerical calculations of static and dynamic parameters. The validation of the FE model and existing SHM system was carried out by the field load testing (Static and dynamic) of the bridge. Further, this study tries to fill the research gap in the area of automatic FE model generation by using a novel methodology that can generate a BIM-based FE model using Visual Programming Language (VPL) scripts. This script can be exported to any FE software to develop the geometry of the FE model. Moreover, the SHM devices are deployed to the Building Information modelling (BIM) model of the bridge to generate the BIM-based sensory model (as per the existing SHM system). In this way, the BIM model is used to manage and monitor the SHM system and control its sensory elements. These sensors are then linked with the self-generated (Internet of Things) IoT platform (coded in Arduino), developing a smart SHM system of the bridge. Resultantly, the system features visualisation and remote accessibility to bridge health monitoring data.

## Introduction

Structural health monitoring (SHM) of bridges has been the subject of interest for many engineers and academicians for a long time. Numerous studies have been conducted in the past to improve SHM systems^1,2^. Recent developments in this study area suggest that the integration of Building Information Modelling (BIM) technology with the SHM system is the need for advanced technological solutions for bridge health monitoring^3–8^.

SHM is the process of monitoring and measuring the structural response in real-time, to detect anomalies in the early stages of damage in structures^9^. The recent trends in the bridge industry involve the development of health monitoring procedures which can maintain the operability and improve the life span of the bridge^10^. The challenge lies in the proper installation and validation before functional use^11–14^. Authentication of the newly installed SHM systems is extremely important for the start of their operational life^15^. Besides this, calibration of the SHM system is required which can be ensured by field load testing of bridge. It results in reliable information on the serviceability and performance parameters of the SHM system. Considering this fact, load testing (static and dynamic tests) of bridge has been performed in this study.

Bien and Kaminski et al. correlated the full-scale test data with analytical models and showed good agreement using static and dynamic tests which yield the maximum vertical displacement of the spans^16^. Lantsoght and Hordijk et al. elaborated the use of diagnostic and proof load testing of RC bridges with their current use and defined the areas requiring future work^17^. Innocenzi et al. discussed the importance of an extensive experimental program (static and dynamic tests) for the proof testing of cable-stayed bridges with the goal of creating a digital twin model for health monitoring purposes^18^. Dynamic testing requires more attention from bridge engineers as it deals with the effects of dynamic factors (moving vehicles, wind, and earthquakes) on the bridge. Thus, many research works targeted the evaluation of the structural modal parameters, for example, time period, resonance frequency, free vibration frequencies, and their respective modes with the corresponding damping ratios^19–23^. Effective utilisation of the load test results involves the comparison of the measured values with the ones calculated in Finite Element Analysis (FEA). If the difference between measured and calculated values is not significant, there is no need to update the FE model. Otherwise, an update of the FE model is required. Both linear and non-linear FEA analyses are popular across the board to carry out the said comparison, therefore, the results obtained from many load tests have focused on verifying bridge provisions of the design codes, procedures, and FEA models^24–28^. Nguyen and Gerges et al. used FE modelling and truckload configurations to predict load limits accurately and reliably and also attempted to monitor bridge health state, load capacity, and aging of a structure^29^, especially in the case of diagnostic load testing.

Generation of the FE model can be performed using direct or indirect integration methods. In the case of bridges, indirect integration methods are found to be more appropriate. These methods can be implemented using a Visual Programming interface, which is based on functional blocks connected in a specific order to perform desired tasks, including mathematical operations, creating, and manipulating geometries, as well as exchange data between the BIM environment and other types of engineering software. VPL has already been used successfully in the automatic compliance check procedure^30,31^, structural optimization^32^, and life cycle sustainability assessment^33^.

The performance of the SHM system majorly depends on the efficiency and robustness of selected sensors^34^. The most commonly employed sensors in SHM systems are LVDTs, strain gauges, accelerometers, and Liquid Levelling Sensors (LLS). All these sensors correspond to the measurement of several factors and quantities that help to assess bridge performance and detect possible anomalies. One of the basic parameters is the strain, measured by robust devices such as vibrating wire strain gauges^35^. In addition to this, the measurement of vertical displacement is also an important indicator for the health evaluation of bridges. The most suitable and promising devices for this kind of measurement are LLS, which can overcome the problem of lack of reference points and provide the most accurate results^36,37^. Other factors include linear deflection measured with Linear Variable Differential Transformers (LVDT)^38^ and angular displacement measured using inclinometers^39^. Vibration monitoring and measurement of dynamic parameters is also an integral part of the bridge SHM system for which MEMS accelerometers offer the best services quantitatively^40^. Weather monitoring stations are also an integral component of the SHM system. These stations are usually installed at the center span of the bridge along with temperature, humidity, anemometer, and barometric sensors.

Naraharisetty et al. discussed the recent developments in the SHM system which are bringing all SHM devices to one platform while linking them with cloud-based servers^41^. Mahmud et al. discussed further benefits of this technology as it is very effective in real-time health monitoring with the advantage of quick data retrieval ability^42^. Further, Natasha et. al., has developed a novel open-source framework, which not only helps to visualize the sensor's data and automatically performs real-time data processing with a frequency of every 2 s^43^. To add technological advancement to this domain, Internet of Things (IoT) technology can be applied to the SHM system for its design, integration with other technologies, modifications, requirements on electronics and energy supply, signal processing, and data evaluation using fog and cloud deployments which can be integrated using BIMification approach^44,45^.

BIMification can be defined as the use of BIM methodologies in any area where BIM can execute a job smartly through cloud-based platforms while controlling and monitoring the system. It plays an important role in integrating sensing technology with the models using a cloud-based solution which is good for automation^46–49^. Using the BIMification approach, all sensors are embedded in the BIM model and their link is established to the cloud-based data platform. In this way, the recording, monitoring, evaluation, and visualization of SHM data can be performed remotely or on-site^8,49,50^. Furthermore, developments in this research domain can scale these integrated BIM models to Virtual Reality (VR)^51^, Augmented Reality (AR)^52^, and Mixed Reality (MR)^53^ platforms through which SHM data can be better visualised using smart cyber-physical devices even on-site.

**Figure 1:**
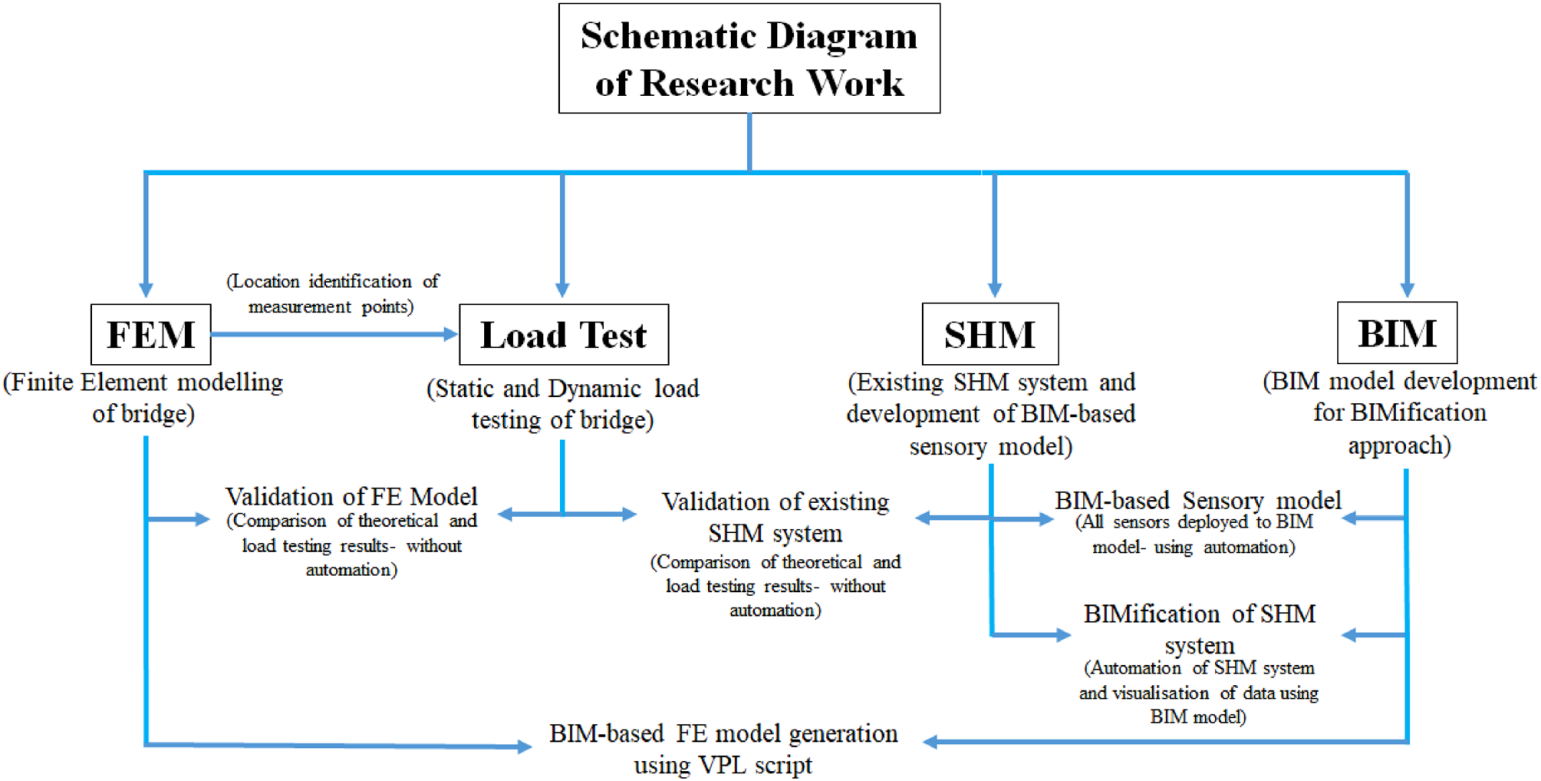
Schematic diagram of research work.

The scope of this research work involves four major research areas, which are interlinked to achieve the aim of this study as shown in Fig. 1. It spans the validation of the existing SHM system using field load testing, FEA of the bridge, the development of a BIM-based FE model, and BIMification of the existing SHM system for its automation and control. Here the term “automation” involves the integration of bridge SHM system with the BIM model, so that a 3D model can directly have access to bridge health data in real-time which can be automatically monitored, visualized, and interpreted. It further involves the real-time monitoring of the service status of the SHM system.

The structure of the bridge with the SHM system was traditionally designed before the implementation of the BIM methodology. Thus, the proposed approach, called BIMification, can be used in the future when modeling new bridges with SHM systems using the BIM environment. As a result, the BIM model could be used to manage and maintain the SHM system and even to control its sensory elements. In addition, by combining the SHM system with a cloud platform, users can visualise the recorded signal data in connection with the bridge's Asset Information Model (AIM). Since most commonly used BIM tools still have numerous limitations in modeling bridges' complex geometry and structure, a methodology for the parametric generation of the FE model from the BIM model is developed in this research work. A proprietary script created in the open Dynamo Visual Programming environment is used. This tool allows the generation of a model for any FE analysis software.

## Experimental program

**Figure 2:**
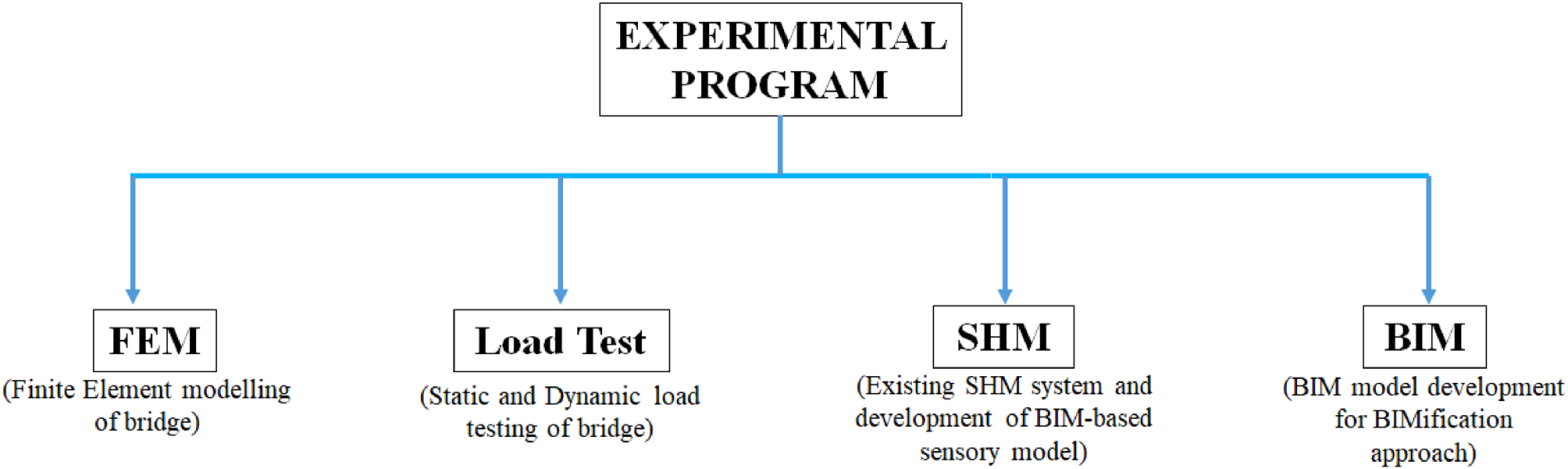
Experimental sketch of the research work.

The experimental plan of this research, as shown in Fig. 2, involves the field load testing of the bridge for the verification of the numerical model and existing SHM system of the bridge, FE analysis of the bridge to evaluate bridge response theoretically and its comparison with load test results, BIM modelling of the bridge for the development of BIMification approach, and visualisation of existing SHM system with the complete details of installed sensors.

### Description of the bridge structure

A newly constructed bridge along National Highway 75 in Poland was selected for this study. This bridge consists of a four-span, continuous extradosed structure with a box girder superstructure made of C60/75 concrete. The bridge spans are 100.0,200.0,200.0 and 100.0 m, making a total of 600 m.

**Figure 3:**
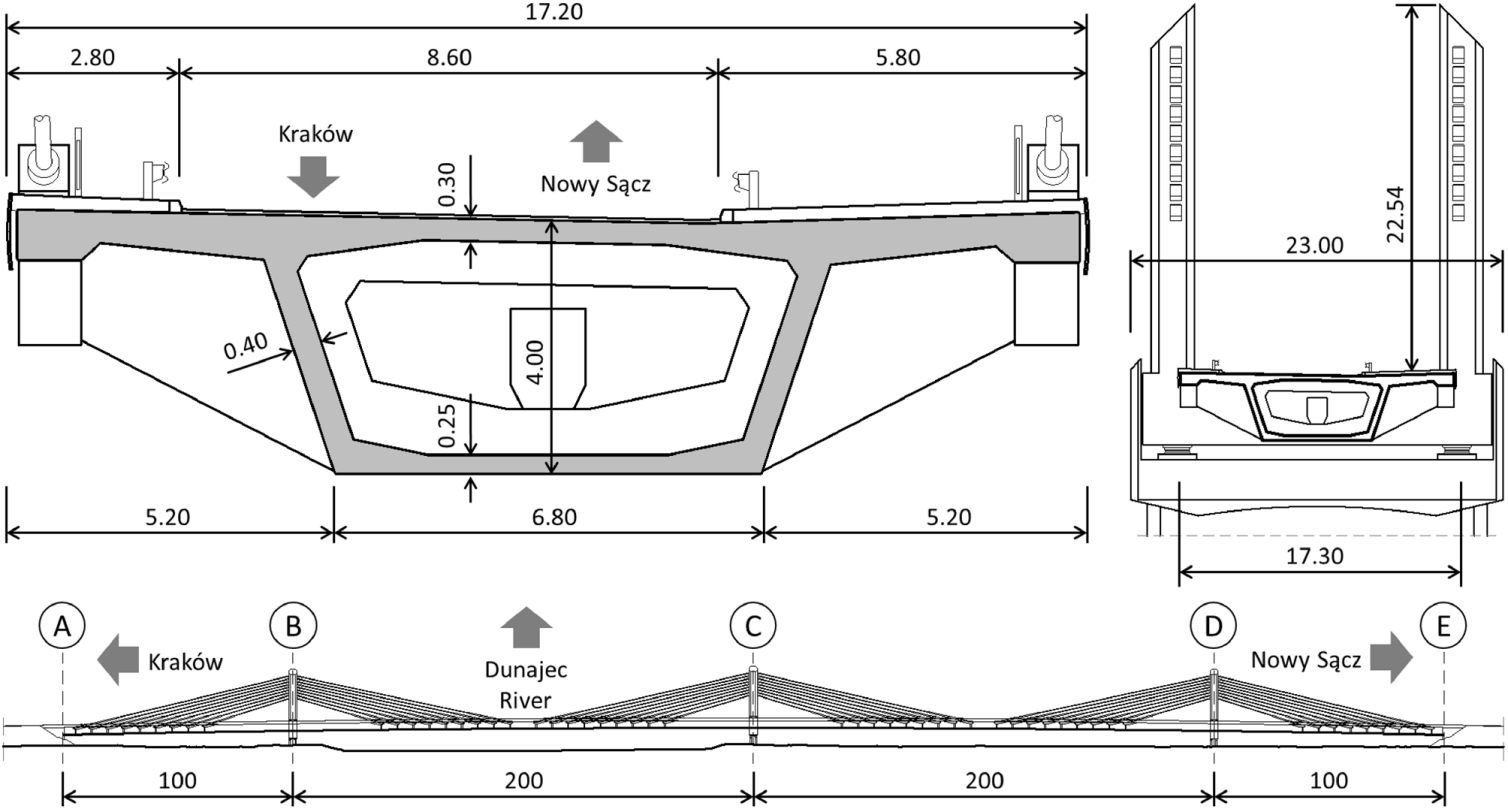
Bridge cross-section and spans layout.

The bridge was designed following the LM1 load model according to the EC1 standard^54^ (with the adaptation coefficients α = 1.00) and for class A according to the PN 85/S standard^55^. The overall width of the structure in the span cross section is 17.68 m and 23.0 m at intermediate supports. A dual carriageway road, 8.60 m wide (between curbs) runs through the structure, there is also a 4.0 m wide pedestrian and bicycle lane. The layout of the bridge with complete detailing of its superstructure and cross-section is shown in Fig. 3.

### Finite element analysis of bridge

**Figure 4:**
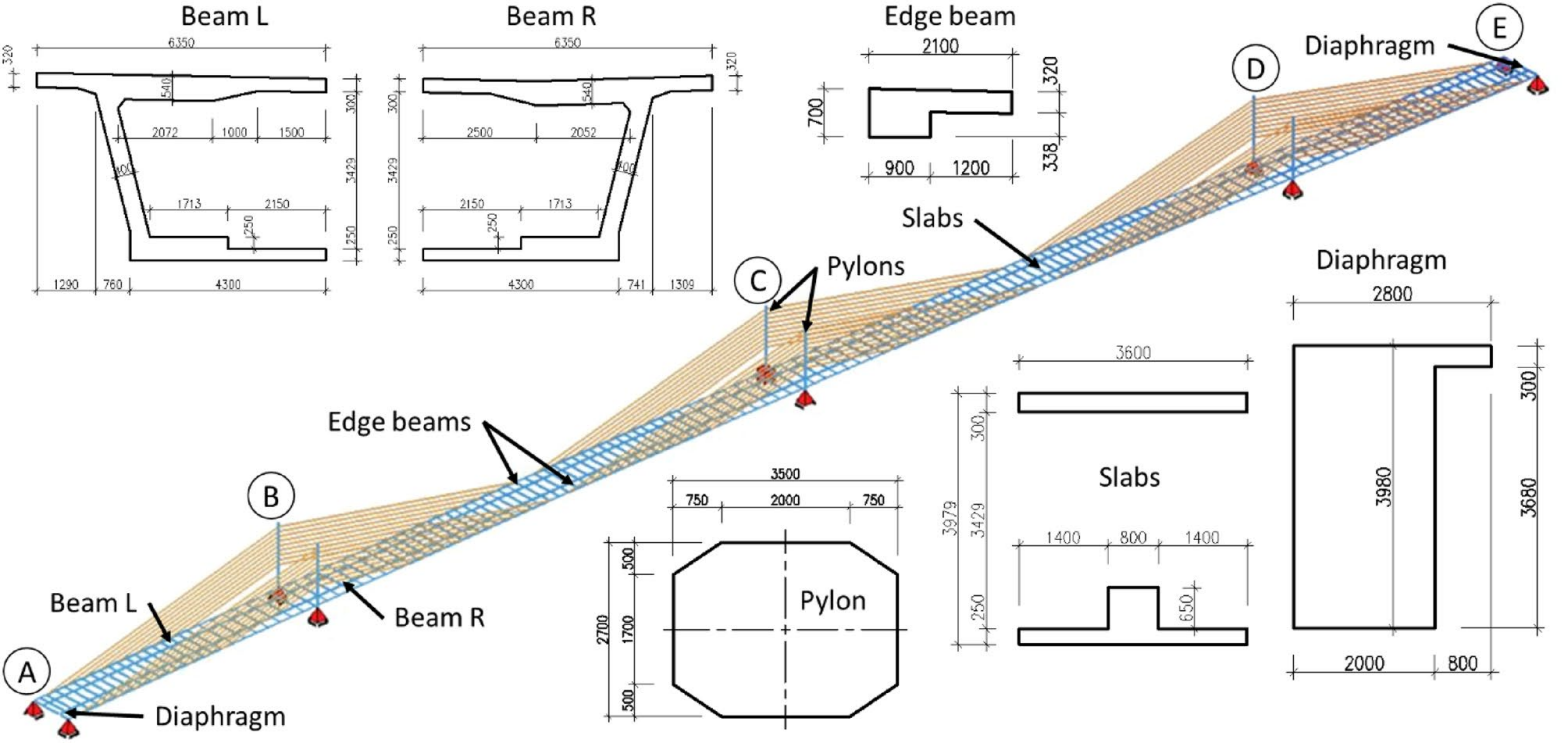
FE model with a basic cross-section of the bridge deck, box girder beams, pylons, and structural elements of the bridge.

Static linear analysis is carried out to calculate internal forces and span displacement. These parameters are required in this study to provide locations of measurement points for the field load testing. Further, these results will be considered as theoretical calculations to compare with the experimental ones (results of load testing). For this purpose, a linear elastic model of the bridge is developed as a shell and line element. The geometric characteristics of the girders and other structural components e.g., pylons were adopted in accordance with the geometry shown in Fig. 4. The geometry of the FE model is developed by dividing the bridge deck into two beams mentioned as Beam-L and Beam R in the Fig. 4. Moreover, the geometry includes upper and lower slabs of C60/75 concrete which are part of bridge deck. Further, the bridge deck is held by the edge beams on both sides of the deck. Similarly, diaphragms are also the part of bridge geometry that helps to resist the lateral forces and transfer loads to the support. The values of the torsional moment are calculated for the entire box section, throughout the girder, for ease of managing the geometry of the structure. The cross-sections of the cables are reduced to a circular cross-section having a diameter resulting from the total cross-sectional area of ​​the strands. In the design, the calculation model takes the standard parameters of the concrete elasticity modulus into account, according to EC2^56,57^.

### Loading regime

Static and dynamic load testing of the bridge was performed in this research. Using the Finite element model, calculations were made to determine the values ​​of bending moments in the box girder to define the load test patterns and location of measurement points. Further, the results of the load test were compared with the FEA results to validate the FE model.

#### Static load testing of bridge

**Figure 5:**
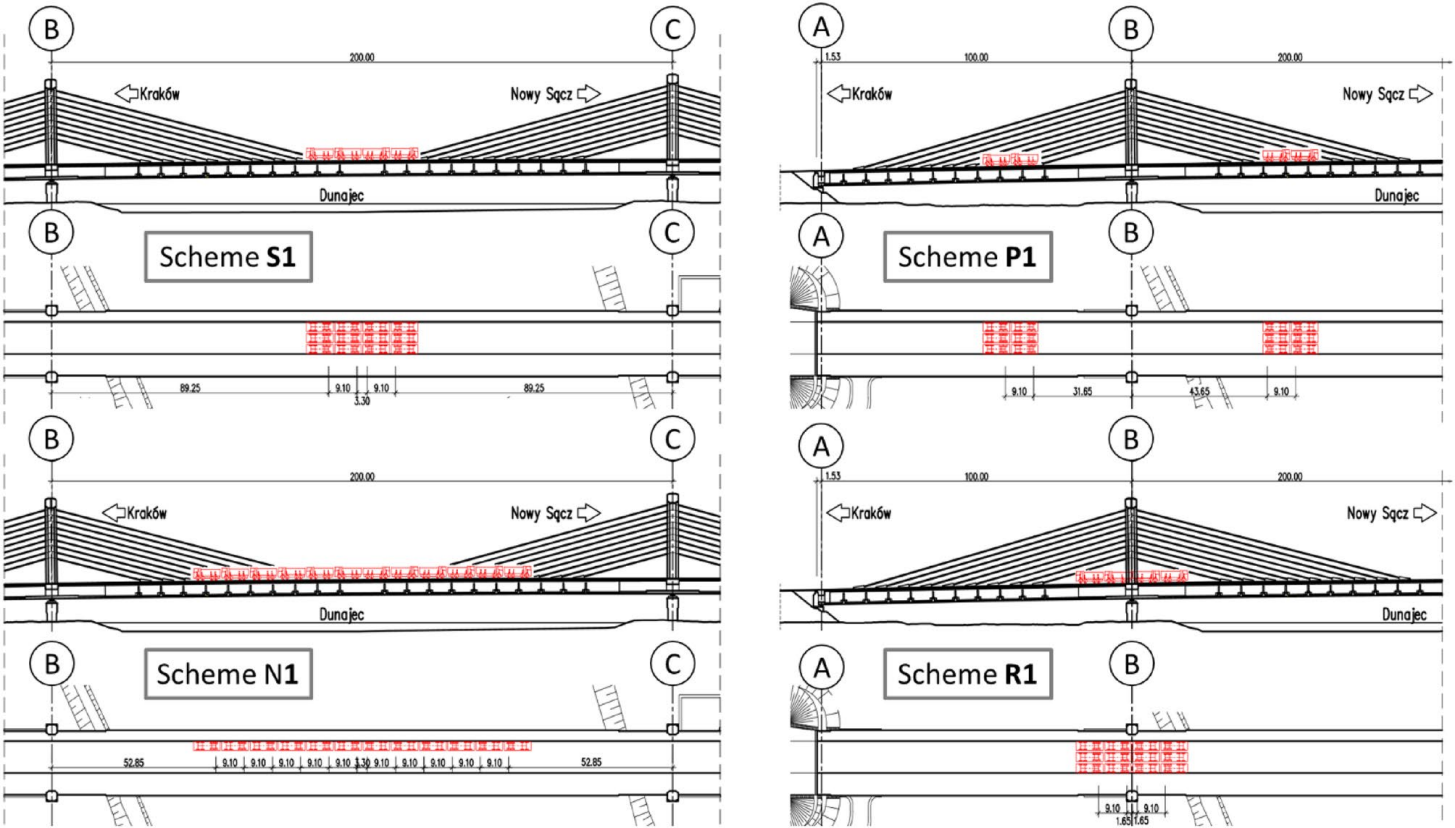
Truck arrangements in the four representative static load test schemes.

**Figure 6:**
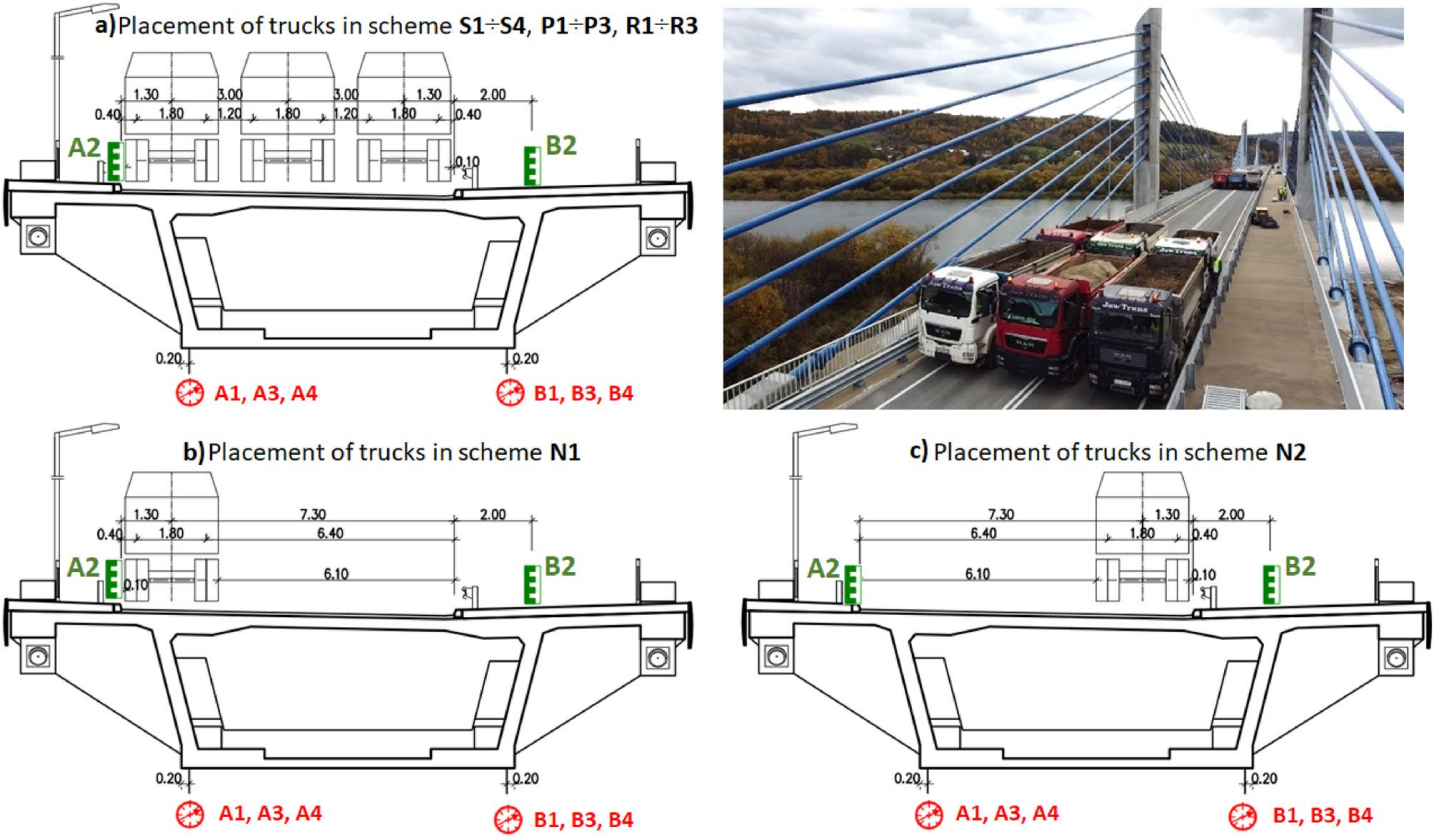
Transverse arrangement of the load test trucks and location of measurement points.

Based on the numerical calculations, twelve load tests, designed to examine the static measurements of the newly constructed bridge in each span, were performed. These tests were performed to check the transverse load distribution and torsional stiffness of the structure. These tests included four basic span tests (S1-S4) selected on the basis of the maximum span load condition, three support tests (P1-P3) selected on the basis of the maximum support cross-section load condition over intermediate supports, three support reaction tests (R1-R3) causing maximum reactions to intermediate supports and two asymmetrical span tests (N1 and N2). As a representative, one scheme of each test is shown in Fig. 5. This figure highlights the placements of trucks along the full length of the bridge. For the scheme “S” twelve trucks were placed at the center of each span, for “P” four trucks on each side of the support at an equal distance, for “N”, twelve trucks at the center of the bridge, and for “R” eight trucks at the axis of the support. The loading arrangement of the bridge along its cross-section is shown in Fig. 6. The truck placement details for schemes S, P, and R are shown in Fig. 6a), for scheme N1 in Fig. 6b), and for N2 in Fig. 6c). The weight of each vehicle was around 32 tons with ± 5% load variation margin giving a total weight of 384 tons.

During the static load test, the displacement of the box girder was measured at two points of its cross-section (under the webs) in the loaded spans, and the settlement of the adjacent supports was also measured under load. Displacement of the girders in spans one, three, and four was measured with a dial gauge having a resolution of 0.01 mm. The measurement in span two was carried out using precise leveling from the top, due to river flow underneath, having a resolution of 0.05 mm. Measurement of support settlements was performed simultaneously with the measurement of deflections.

**Figure 7:**
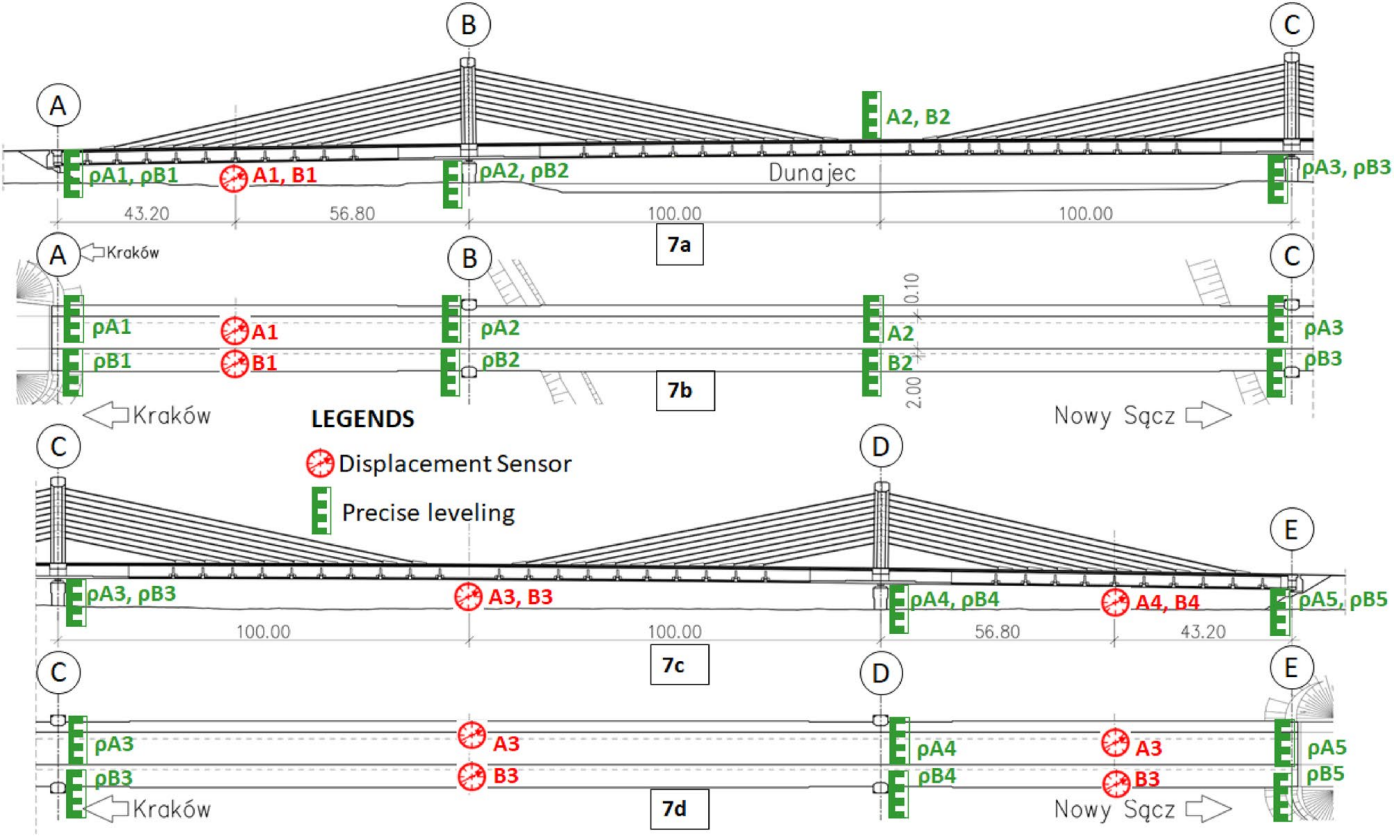
Location of measuring points and installed sensors during the static load test.

Displacement sensors and precise levelling (at each support) were used to measure the bridge displacements during the static tests. These sensors were placed according to the placement of trucks (static loads), and accordingly, measurements were taken. The markings of the measurement points and the location of these sensors are shown in Fig. 7. The displacement sensors were placed on each beam (L and R) at the center of each span and are highlighted in Fig. 7b and d. Similarly, the precise levelling sensors were placed at each of the support on both sides as shown in the cross sections of Fig. 7a and c. The first series of measurements were done in an unloaded state. Subsequent series (not less than three) were carried out at regular intervals (every 10 min.) until the movements stabilized. The stabilisation of displacements is understood as a situation where the difference between the indicated and its previous displacement does not exceed 1% of the measurement increment. At this point, the structure was unloaded, and a series of readings were made again.

#### Dynamic load testing of bridge

Dynamic load testing was carried out to identify the dynamic properties of the bridge superstructure. During the test, modal parameters (natural frequency, mode shape, and damping ratio) associated with basic modes of vibration were identified. Identification of modal parameters was performed on the basis of acceleration signals processed using the Operational Modal Analysis (OMA) framework. SSI-COV algorithm was implemented in the modal identification.

**Figure 8:**
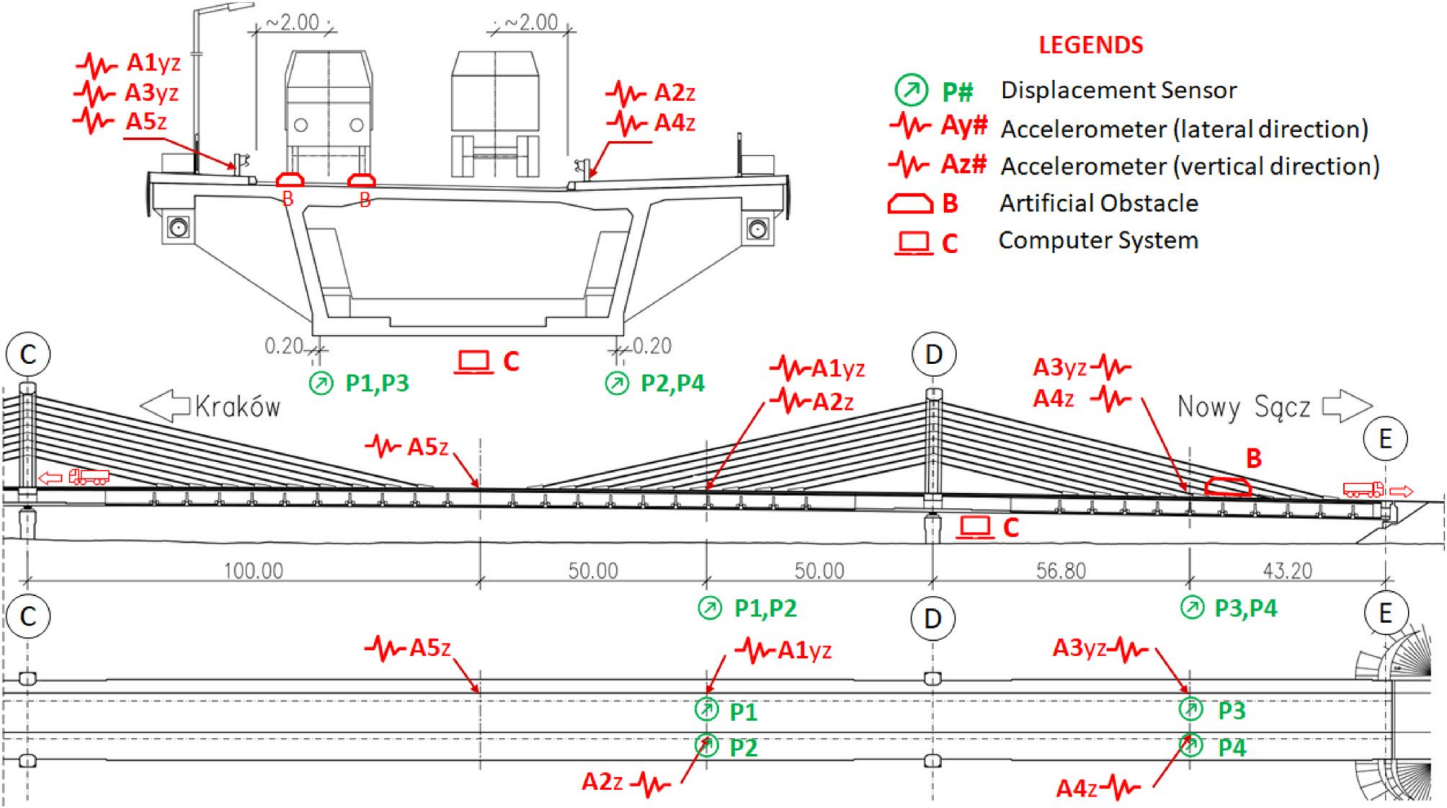
Arrangement and placement of sensors along the bridge cross-section used in the dynamic load test.

To perform the dynamic load testing, Peltron inductive linear displacement sensors (LVDT) were used to record dynamic displacements of the bridge and were placed at the marked locations in Fig. 8 (green colour). LVDTs were attached to the bottom slab of bridge decks and measurements were taken beneath the bridge. Similarly, PCB Piezoelectric high sensitivity accelerometers (IEPE) were used to record vibrations of bridge on each side of the roadway with the location marked in Fig. 8. (red colour). The test results were recorded electronically as time signals of vertical displacements and vertical (z) and transverse (y) accelerations. The measurement system also included a laptop computer and Siemens LMS SCADAS Mobile data recorder as shown in \* MERGEFORMAT Fig. 8. Further, an artificial obstacle was placed at the route of the truck to produce some excitation for the OMA of the bridge. This obstacle is shown in the span ‘DE’ of Fig. 8.

### Overview of the bridge SHM system

The SHM system of the bridge was planned to continuously monitor the technical condition of the structure. Considering the need, the SHM system was designed for the installation of sensors at selected measurement points. The major purpose of this system was to assess rheological phenomena, assessment of structural behavior of the bridge under operational loads, observation of the operationality of bridge box girders and suspension system, identification of structural damages, and monitoring of loading conditions of the bridge. In this way, the SHM system can perform deformation measurements, synchronous dynamic measurements (Dynamic deflection and accelerations), structural response measurements under the operational loads, and monitoring of temperature, humidity, and wind effects on the structure.

To monitor the different parameters of bridge health certain devices like Vibrating wire strain gauges, Liquid Levelling Sensors (LLS), LVDTs, MEMS accelerometers supplemented with weather monitoring control stations and a Data Acquisition System (DAQ) is part of this SHM system. Their complete details, types, and location points are enlisted in Table 1.

**Table 1:** Details of installed sensors on bridge.

Sensor name	Measurement parameter	No. of sensors	Location in Span#1 (Fig. 9)
Embedded wire strain gauge (WSG)	Deformation in pylons and slab	28	03, 05
Liquid Levelling Sensors (LLS)	Vertical displacement	15	02, 03, 04
LVDTs (LVD)	Displacement at supports	10	A, B
Inclinometers (INC)	Angular displacement	20	02, 03, 04 & B
MEMS accelerometers (ACC)	Acceleration monitoring	28	03 & 1st, 3rd cable
Meteo station (MET)	Temp, Humd, and wind	01	Center of bridge

**Figure 9:**
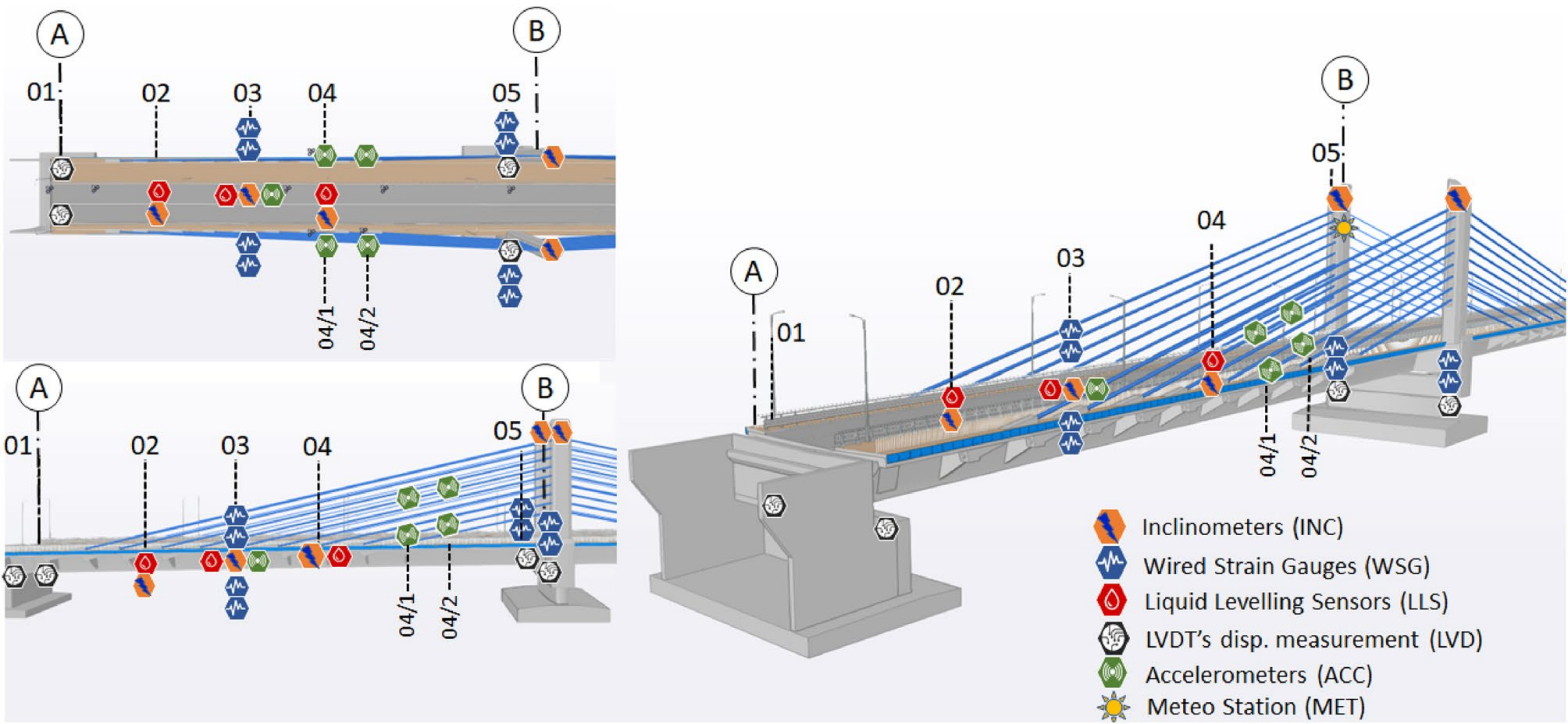
SHM model of the bridge.

The above-mentioned sensors are contributing to the SHM system of the bridge, which is the major focus of this study. The BIM model of the bridge is selected to show the location of these sensors. For simplicity, the BIM-based SHM system for one span of the bridge is visually elaborated in Fig. 9, which will be further used for the automation of the SHM system.

### BIM model development

**Figure 10:**
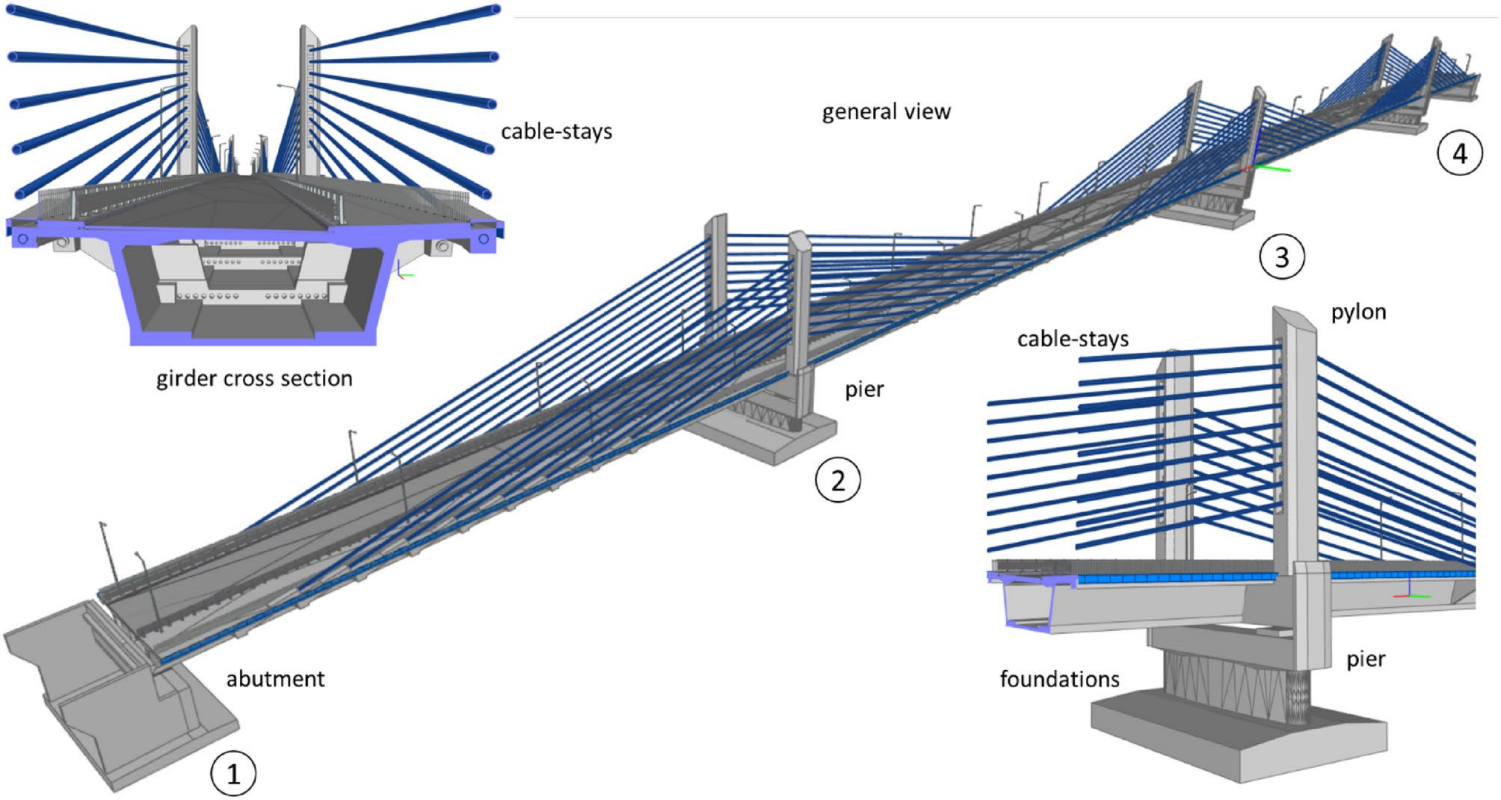
BIM model of the bridge.

The viability of BIM technology has been explored in this research and accordingly, the BIM model of the bridge was developed using Autodesk Revit software (Fig. 10). All the families of structural elements were imported from the Revit data pack and used by importing actual details of materials and accessory elements.

This model provides a base for the novel solution proposed in this research. These solutions helped to generate an accurate parametric FEM model, as well as suggested efficient visualisation of real-time sensor data. Visualisation of SHM data is very handy for bridge inspectors as they can use the suggested application during bridge inspection and see the real-time condition of the bridge by interpreting the information provided by sensors regarding the bridge's health. Integration between BIM and FEM models ensures a coherent database with the ability to generate and update structural models in an automatic or semi-automatic way.

## Results and discussions

### FE analysis results

Using the structural model, calculations are carried out to determine the values ​​of displacement and the maximum bending moments in the spans and cross-sections of the box girder. These values are further compared against the code limits and measured values of load tests (measured in Section "Static load testing of bridge"). The results show that the bridge has sufficient moment capacity. The calculations ignored the influence of axial forces due to their low values ​​and normal stresses in the considered cross-sections. Based on our own experience, the model results could be enhanced if we use more realistic concrete strength (instead of the standard value) as per the real situation (e.g., measurements on the existing bridge). Moreover, the results of the static analysis indicate a slightly higher flexural stiffness of the structure, necessitating the need for the modification of the modulus of elasticity.

The modulus of elasticity of concrete is the measure of concrete’s stiffness and is an important factor to calculate the flexure stiffness of the structure. It needs to be quantified as per the required reduction factor, described according to EC. This modification is also considered during the development of the FE model, thus FE model with the corrected value is considered in this research. This correction is described in the section below.

#### Modification of concrete Modulus of Elasticity as per EC^54^

The dependence of the modulus of elasticity of concrete on the type of aggregate used is taken into account in EC-2 by the coefficient *α*_E_. The modulus of elasticity *E*_*cm*_ for concrete is thus calculated by multiplying the values ​​by the coefficient *α*_*E*_. In the case of used aggregate, the *α*_E_ coefficient is 1.2. Considering the influence of air entrainment in the analysed structure, *α*_E_ is finally assumed to be equal to 1.2. Considering the concrete strength tests after 28 days of curing and the type of aggregate used, the forecasted modulus of concrete elasticity as a function of age can be determined based on the formula:

1 $$E_{cm} \left( t \right) = 22 \cdot \left( {\frac{{f_{cm} }}{10} \cdot e^{{s \cdot \left( {1 - \sqrt{\frac{28}{t}} } \right)}} } \right)^{0,3} \cdot \alpha_{E} \quad \quad [GPa],$$

where: *fcm* is the average 28-day compressive strength of concrete in [MPa], *s* = 0.25 is a factor depending on the type of cement (CEM II / AS 52.5 N), *t*—is the age of concrete ≥ 7 in days, *α*_*E*_ = 1.2 is a factor as discussed above.

**Figure 11:**
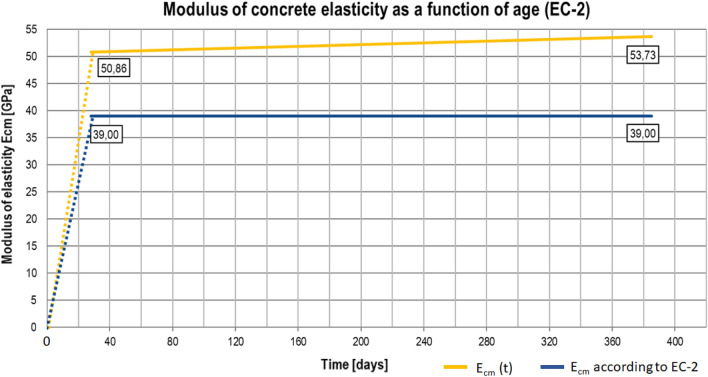
Modulus of concrete elasticity as a function of age (EC-2).

The calculated modulus of elasticity *E*_*cm*_ of the concrete is found to be higher than the E_cm_ as per EC. The comparison of both of these values is presented in the graphs of Fig. 11, showing that the resulting increase in the stiffness of the structure can be considered by reducing the theoretically calculated deflections by a reduction factor of 0.73 (Table 2).

**Table 2:** Reduction factor of predicted modulus of elasticity according to EC2.

Age of concrete	Average strength	Characteristic strength	Concrete class	E_cm_	E_cm_ as per EC standard	Reduction factor
t	f_cm_ (t)	f_ck_ (t)	[–]	[–]	[–]	E_0_/E_cm_ (t)
[days]	[MPa]	[MPa]	[–]	[MPa]	[MPa]	[–]
385	103	99	C 60/75	53.7	39	0.73

For the purpose of updating the FEM model, the change in stiffness EI of concrete spans was taken into account by introducing parameters consistent with the concrete mix used. According to the EC standard^54^, the modulus of elasticity of concrete depends on the function of age, type of aggregate, and cement. Knowing the composition of the concrete mix and the results of concrete compressive strength tests after 28 days of curing, the change in the modulus of elasticity was estimated. The obtained value was corrected in relation to the standard value specified by the designer. This approach is acceptable in the absence of laboratory tests of the modulus of concrete elasticity.

#### Validation of the FE model

FEA can be verified by the results of field load testing. The FE model was validated by comparing the results of the field test with the numerical output, especially displacements. These values are further compared against the code limits and measured values of load tests (Table 3). Further, the consistency of the stiffness values is also checked. Thus, a comparison of permanent and total deflection is carried out and checked against the standard condition of not exceeding the level of 10%^29,56^. In this way, load-testing results are justified as a source of FE model updating (Section "Static load test results").

**Table 3:** Maximum deflections of the box girder in selected load schemes [mm].

Static load scheme​	S1​		S2​		N1​		N2	
Span​	1​		2​		2		3	
Measurement point​	A1​	B1​	A2​	B2​	A2​	B2​	A3​	B3​
Total displacement, *U*_*t*_	29.1​	28.2​	119​0.3	117.9	101​0.0	95.6​	93.5​	94.6​
Permanent Deflection, *U*_*p*_ ​	− 0.1	− 0.3​	4.3​	4.8​	2.1​	2.1​	− 0.2	− 0.2​
Elastic Deflection, *U*_*e*_	29.1​	28.2​	115.1	113.1​	98.9​	93.6​	93.5​	94.6​
Theoretical Deflection, *U*_*d*_	34.3	33.05​	127.2	124​0.6	109.4	104.4	106.2	108.1
Permanent/Total, $p$	0%	0%​	4%​	4%​	2%​	2%​	0%​	0%​
Elastic/Theoretical, *U*_*d*_ $(k$*)*	85%	85%​	90%​	91%​	90%​	90%​	88%​	88%​
Average	86%				89%			

### BIM-based FE model development

The final changes in the FE model can be incorporated according to the results of the load tests. In this way, updating the FE model requires some iterative steps where it becomes necessary to make changes in the geometry of the FE model and the properties of elements or materials. In most of the software, the said job is cumbersome and consumes a lot of time. To overcome this issue, an integrated FE model generation technique is developed in this research. It uses BIM model as a source file and transforms its geometry into the topology of the FE model. The future direction of this work focuses on the material assignment, load application, and running the FEA using the BIM-based model, which can lead to the comparison of BIM-based FEA results with the traditional FEA.

**Figure 12:**
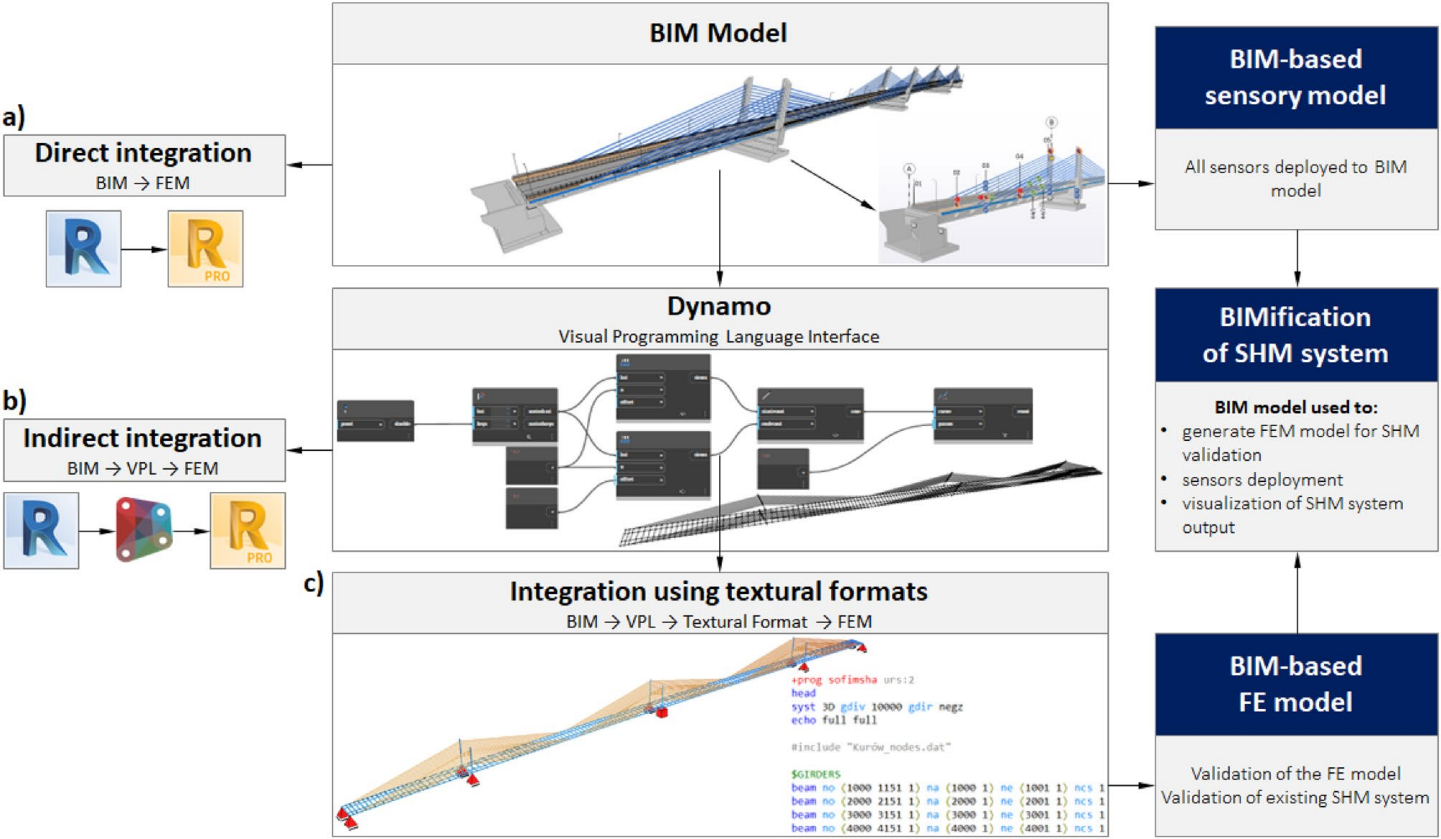
Methods of the generation of the FEM models based on the BIM model.

The generation of the BIM-based FE model can be performed using direct or indirect methods (Fig. 12). Direct integration (Fig. 12a) involves closed solutions provided by BIM and FEM software. Linear elements, including beams, columns, pylons, or cables, can be translated into analytical counterparts created automatically in the structural analysis environment. It is observed that this method generates valid models only when the topology of both the BIM and the FEM models is similar in terms of the number of elements, their shape, orientation, and relations. Moreover, the direct generation of the structural model requires dividing the BIM model into pieces, including elements that are not explicit in the real structure, e.g., longitudinal, and transversal beams. Introducing such a topology in BIM environment, especially for structural analysis purposes, is not a valid approach, as it requires additional, separate, and virtual elements to the BIM model that can disturb its semantics, performance, and usability in other aspects, e.g., quantity take-offs. Therefore, indirect method have been used in this research.

**Figure 13:**
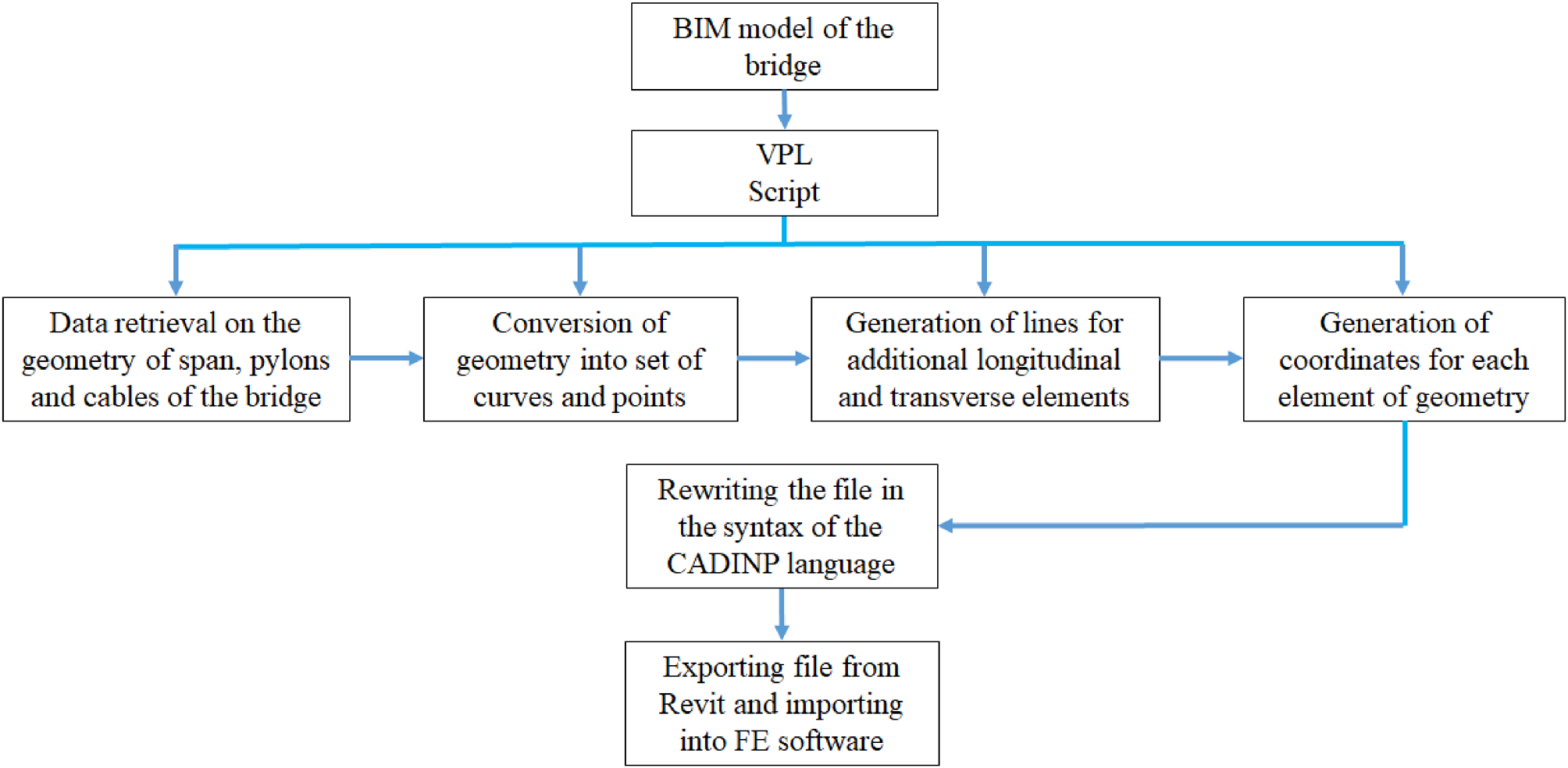
Schematic of BIM-based FE model development.

In this approach, the VPL interface is used to retrieve data on the geometry of spans, pylons, and cables directly from the BIM model and convert them into a set of curves and points, including additional lines for longitudinal and transverse components of the structural model. This geometric representation can then be used to generate FEM models using additional packages in Dynamo (Fig. 12b) or textural formats readable by structural analysis software (Fig. 12c). Using a visual script, a file is generated that contains the coordinates of all the nodes. This file is written in the syntax of the CADINP language used in FE software. The sequence of all the mentioned steps is shown in Fig. 13.

This approach allows using other engineering software while exchanging data between BIM and FEM environment since a generic list of nodes coordinates is the output of the Dynamo script. The output is universal enough to be successfully implemented in other frameworks of data exchange between the BIM model and any FEM software. Using visual programming allows for the creation of parametric models directed by an open and fully adjustable code. The robustness and modifiability of direct methods are limited and depend on the maturity of the software used. Direct methods are usually software-specific and closed implementations delivered as ready-to-use tools in a software interface, defined in compiled and inaccessible source code that cannot be adjusted or extended to perform specific, out-of-scope tasks. In the given example and software, the BIM model topology cannot be directly transformed into the FE model due to inconsistencies in the structure of both models. The single cross-section of the superstructure extruded along the road alignment creates a solid span and would be seen as a single linear element in the FE model. The topology of the span requires divisions into sections of parametric density defined in the open VPL code. Furthermore, two series of 1D elements are created for the left and right sides of the box girder with neglected vertical alignment in the FE model. These basic rules may not be fully covered by direct methods of data exchange, depending on the specificity and maturity of the software. In the case of the bridge FE model, due to the irregularity, complexity, and curvature of the BIM model geometries, direct methods of exchange can give incorrect output. Hence, open indirect solutions are recommended and presented in our approach that can be extended or modified, if required, and give the output that is not limited to specific FEA software. The use of indirect methods also has other advantages. In this way, not only the time is saved but it is also very easy to update the FE model according to the results of the load testing. The openness and transparency of the source code are maintained, which is one of the key features of the BIM methodology, e.g., IFC-based (Industry Foundation Classes) data delivery and exchange. The said approach can be the basis for BIM-based bridge FEA and, in the future, can help designers work on the BIM model to carry out FEA.

### Static load test results

Based on the deflection readings recorded during the test, vertical displacements of the webs are calculated at different positions in the span. Each time, the displacement values are referred to the initial state, before loading. Selected representative results of displacement measurement are listed in Table 3.

The values ​​determined here are subjected to successive stages of loading and unloading of the structure during load tests. So, after completing the measurements, measured total displacements (U_t_) and permanent displacements (U_p_), the elastic displacements or deflections (U_e_) are determined and compared with the corresponding theoretically calculated (based on FEM analysis) values (U_d_). The comparison between them is expressed as a percentage of the difference between theoretical values (FEM) and measured ones. Following standard conditions are applied to carry out these comparisons^56^:

- The ratio of permanent deflections *U*_*p*_ to total deflections *U*_*t*_
  3 $$p=\frac{{U}_{p}}{{U}_{t}}<10\%.$$
- The ratio of elastic deflections *U*_*e*_ to design (theoretical) deflections *U*_*d*_
  4 $$k=\frac{{U}_{e}}{{U}_{d}}<100\mathrm{\%}.$$

-

From the results enlisted in Table 3, it can be observed that the stiffness of the spans is in accordance with the properties of the concrete used. The elastic deflections of the maximum loaded box girder in each span are slightly smaller than the theoretically calculated (amount to 85% and 90%) values. On average, elastic deflections of the tested spans constitute about 86% of the calculated values. Whereas the comparison of permanent and total deflections constitutes from 0 to 6%, therefore, meets the standard condition of not exceeding the level of 10%^29,56^. Such results prove that the stiffness of the spans is consistent with the values of the calculation model, thus validating the FE model of the bridge and showing the sufficiency of existing sensors measuring the static parameters of the SHM system.

### Dynamic load test results

**Figure 14:**
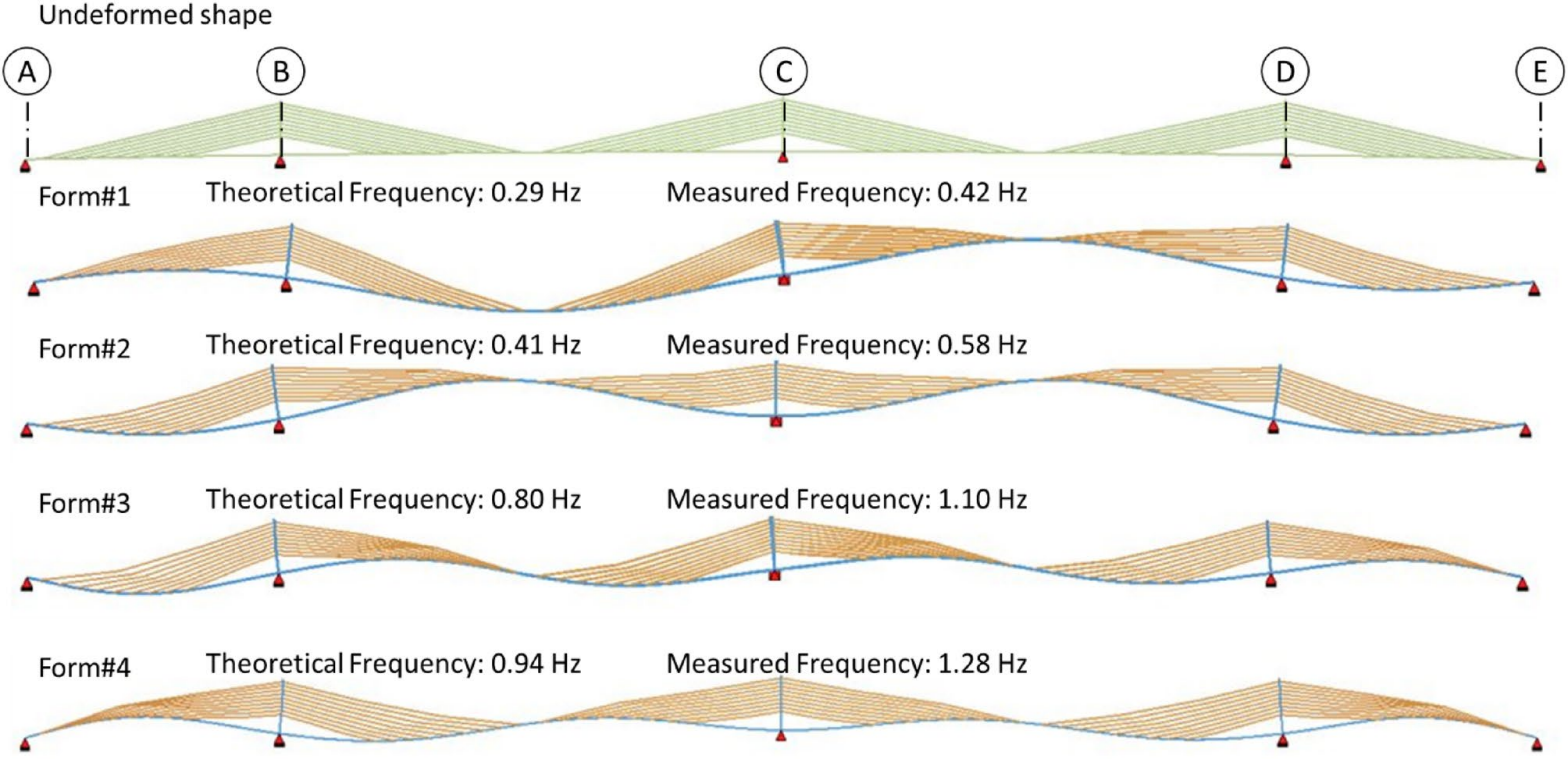
Mode shapes from the FE model, preliminary theoretical and real identified natural frequencies.

**Figure 15:**
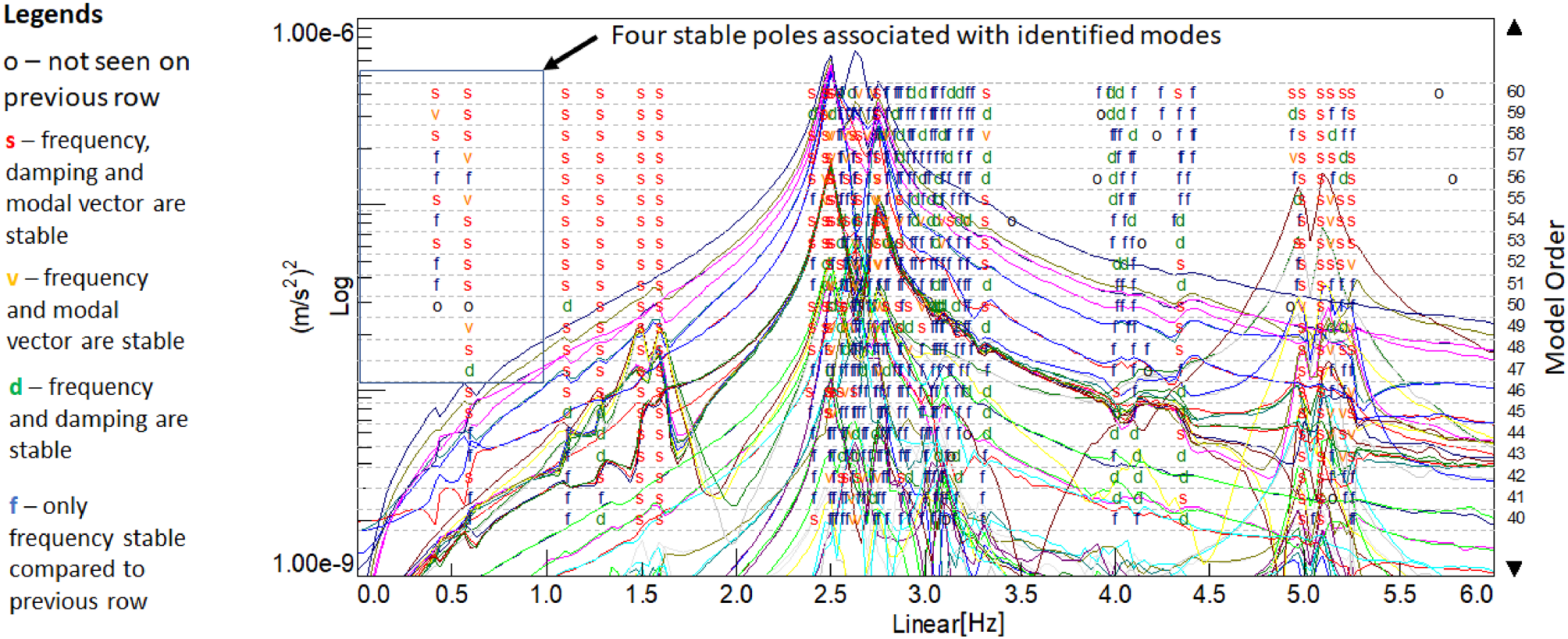
Stabilization diagram of SSI-COV identification algorithm.

Measurements of dynamic load testing have unambiguously identified four natural frequencies which are shown in Fig. 14. In the case of the mode shapes, only FE analysis results give valuable information, so, mode shapes from FE analysis are considered. As observed in Fig. 8, sensors number (only five vertical accelerometers) and placements (only two spans) limit our options to visualize mode shapes from Operational Modal Analysis, thus, only the comparison of frequencies is carried out without a comprehensive comparison of shapes using e.g. CoMAC (Coordinate Modal Assurance Criteria). The lowest identified natural frequency is 0.42 Hz which is higher than the theoretically calculated 0.29 Hz. Even though stabilization diagram (Fig. 15) shows numerous peaks that indicate other natural frequencies, due to their proximity in the frequency domain and the complex shape of the sought forms, unambiguous identification is not possible. Thus, it would be necessary to use a larger array of sensors spread around key locations in a whole superstructure. This conclusion is pointing toward the need for some additional accelerometers with more measurement points at the bridge.

The stabilization diagram shown in Fig. 15 gives better insight into superstructure dynamic properties. It shows the stability of four poles as “f—only frequency stable compared to previous row; d—frequency and damping are stable; v—frequency and modal vector are stable; s—frequency, damping, and modal vector are stable”. Here the model vector is considered between 40 and 60, which highlights the number of modes used to describe Frequency Response Functions (FRFs). It can be clearly seen that the most excited frequencies lay around 2.5 Hz but as stated previously, a relatively small number of sensors have limited mode observability.

Another metric of the dynamic behavior of the bridge is the dynamic amplification factor (DAF), here defined as the ratio of the maximum deflection on a given drive (30, 50, 70 km/h) of one vehicle to the maximum deflection in the same measurement section while driving the same route at a quasi-static speed of up to 10 km/h. The DAF peaked at 1.05 at 70 km/h concluding that the bridge has a small dynamic susceptibility to excitation by heavy vehicles^21^.

**Figure 16:**
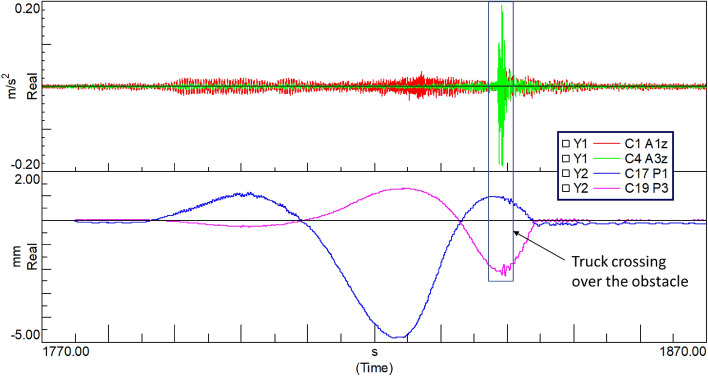
Raw acceleration (top) and displacement (bottom) data recorded during the test ride passing over the artificial obstacle.

The graph of changes in the time domain of displacements (point P1) and accelerations (point A2) is shown in Fig. 16. This graph shows the results of the harmonic analysis, where the vibration spectrum contains peaks revealing the fundamental vibration frequencies. Further, the excitation caused by the artificial obstacles can also be observed as a peak in both of these graphs.

Observation of these results, in the natural frequency domain, shows that the structure has a slightly higher stiffness than assumed in the model. Thus, the trend observed in static tests is also confirmed. As already mentioned, the results of the dynamic tests highlight the need for a greater number of accelerometers and dynamic measurement locations because of the higher difference in the measured and theoretical natural frequencies. Figure 8 shows that only five sensors are used along the cross-section of the bridge. Out of these five sensors, two sensors are measuring both longitudinal and transverse vibrations, two are measuring vertical vibrations, and one sensor measuring lateral. Using a greater number of sensors increases the observability of the higher modes. Therefore, the results, with the given number of sensors and differences in their locations, are not very conclusive in the case of field measurement. Resultantly, the analysis is more reliant on the outcomes of the FE analysis which is recommended in this research.

After the evaluation of load testing results, it can be concluded that the type of sensors measuring the dynamic parameters of the SHM system are sufficing the needs of the existing SHM system whereas there is a lack of the number of sensors, and measurement location for dynamic parameters, therefore existing SHM require more of such devices for reliable monitoring of dynamic parameters.

## BIMification plan of SHM system of bridge

**Figure 17:**
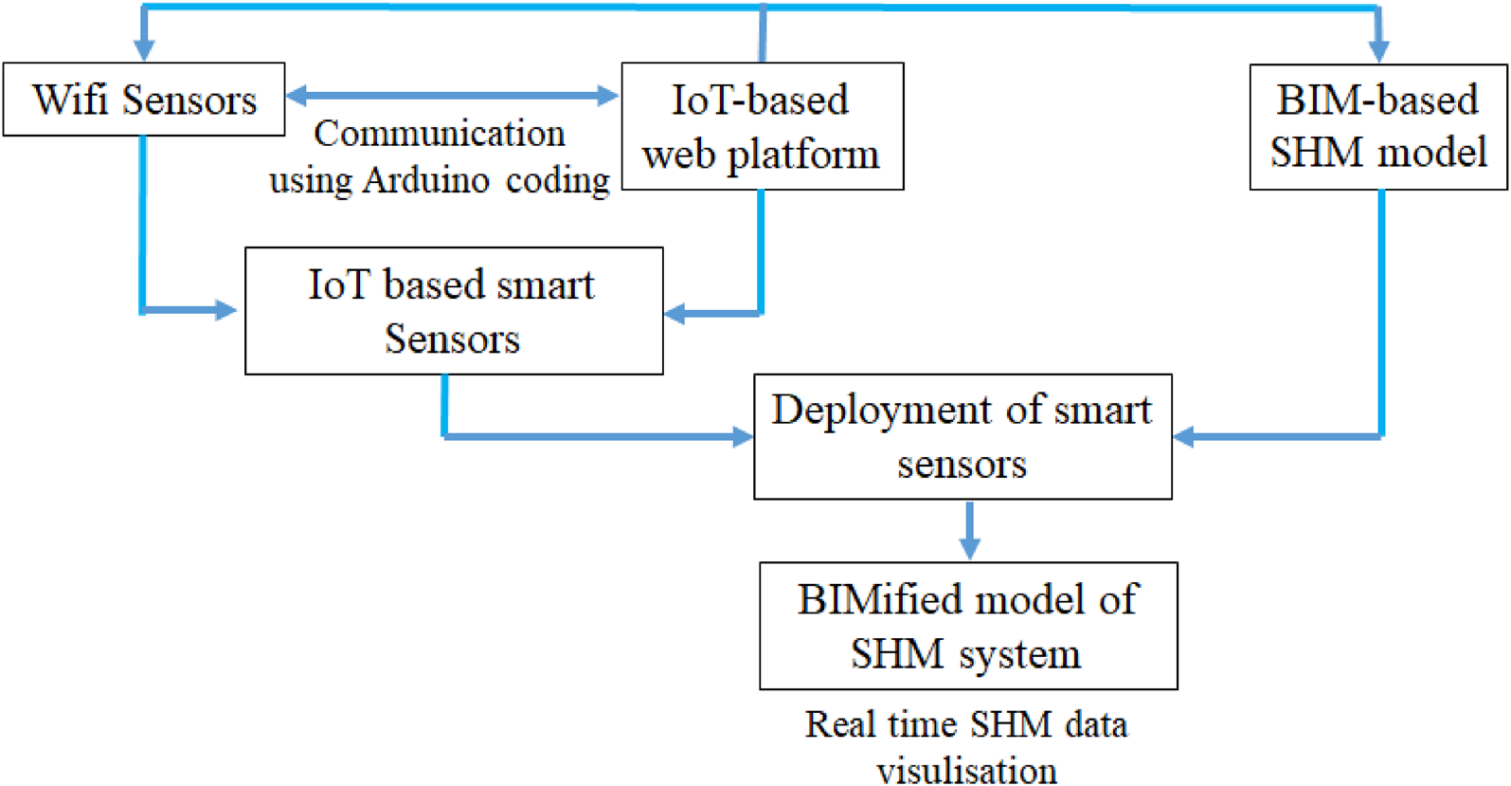
BIMification process of the SHM system.

This section proposes a novel way of linking the SHM system with the BIM model of the bridge so that it can be managed and controlled automatically. This approach is termed BIMification of the SHM system. There are three major components of this BIMification approach: (1) smart sensors (2) an IoT-based web platform (3) a BIM-based sensor model. Schematic of the whole BIMification process is shown in Fig. 17.

**Figure 18:**
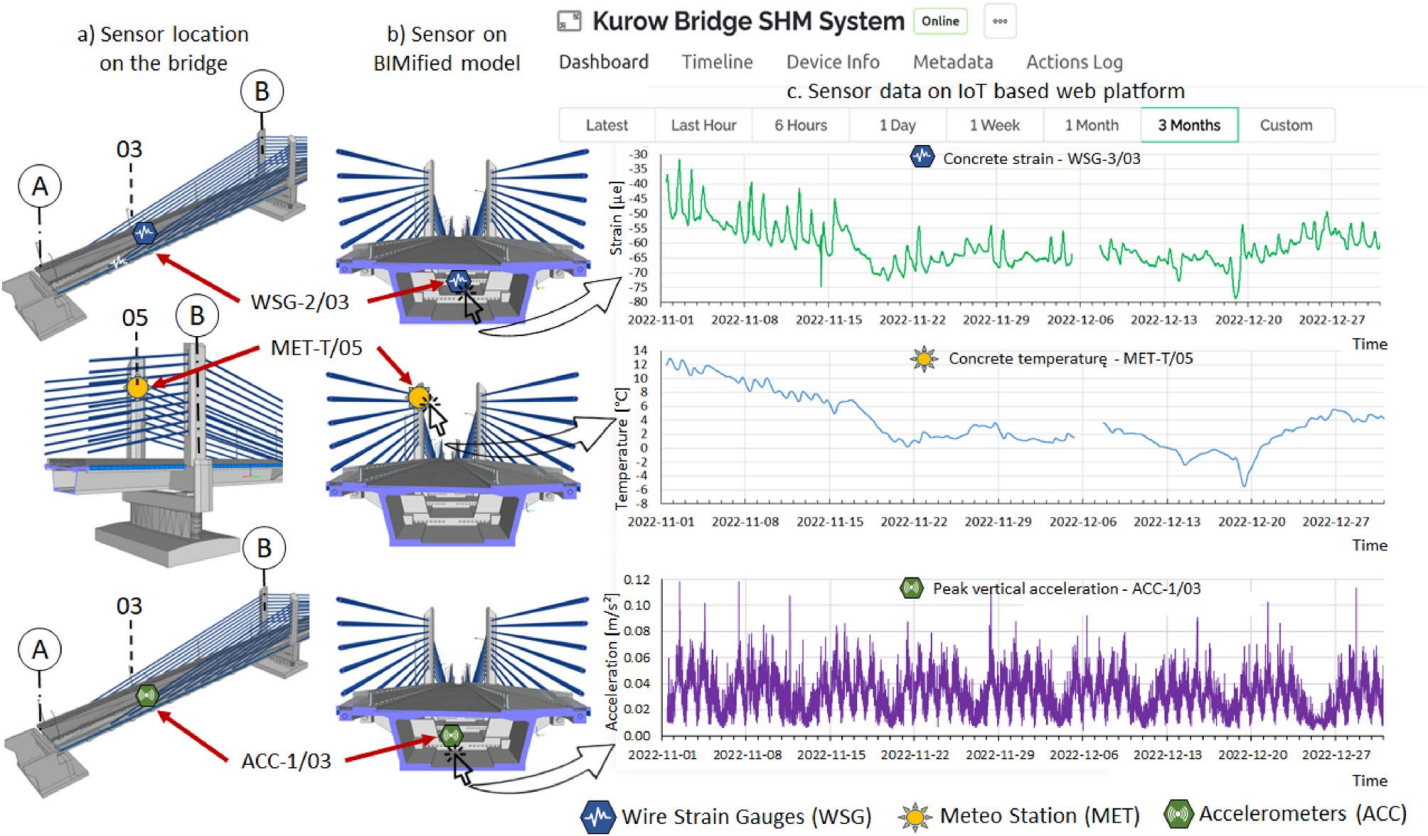
Visualisation of SHM data using a cloud-based platform.

The authors have used three sensors as representative of the bridge SHM system in this research. These sensors include a strain gauge, a temperature sensor, and an accelerometer (Fig. 18). These sensors have special identifiers in the system, showing the abbreviations of their names and location along the bridge. The strain sensor is identified as WSG-2/03 (03 section), the temperature sensor as MET-T/05 (05 section), and ACC-1/03 (03 section). All these sensors were physically installed on the bridge and whole communication between the sensors and web platforms was carried out through an API-based access.

The second most important and powerful tool of BIMification process is the web platform. It is developed using a free version of a web application. This application is available in both smartphone and web versions, but the developer mode is available only on the web version. So, using the web version of the application first, the web platform is developed, and after that communication between the sensors and the web platform is established. To develop this platform, a project is created by the name of the bridge SHM system, where one device having three sensors (strain sensor, temperature sensor, and vibration sensor) is added to the dashboard. On the dashboard, each sensor is created with its own domain, and its measurement features are enabled according to its datasheet. Thus, the measurement parameters of each sensor, i.e., its measurement limits, units, graph axis, and frequency of measurement are manually developed. After developing the graphical interface and measurement scheme of sensors, they are made online by connecting to the web platform. This connection is developed using Arduino IDE codes. One code for all three sensors is developed where API of the sensors and domain of the web platform is embedded. This code is then uploaded to the web application dashboard and communication is established over the Internet. After successfully developing the connection between sensors and the web platform, the sensors under the SHM system are visible online on the dashboard. After this, automation action is enabled for sensors that allow data recording. This initiates the real-time monitoring of the bridge.

The next step is the creation of BIMified sensory model, which is developed by deploying these smart sensors to the BIM model of the bridge. The job is done using the MR application. The web-supported domain is used throughout this process too. The BIM model of the bridge is uploaded to the application platform as the IFC formats or .rvt files, directly through the BIM software. After uploading the model, the locations of the selected sensors according to the layout shown in Fig. 9 are marked on the uploaded BIM model. At these locations, small icons representing each sensor are then generated. All these icons are now the virtual representation of the actual sensors installed on the bridge. Each icon in the BIM environment is developed with several functions. The major task was to develop the URL access for each icon, which is done using the interface of the MR application. This function allows the user to embed the URL or IP address of the web platform dashboard, where recorded data is available in graphical format, so, after developing this communication path in the BIM model, the MR application developed direct access to IoT-based web platforms. Clicking the sensor icon on BIMified model redirects to the sensor dashboard on the web platform where real sensors are sending data in graphical formats. Graphs of each measurement parameter can be seen in Fig. 18. In this way, data with different options (real-time, one hour, one day, seven-day, one month, and three months) can be visualised remotely. Clicking each graph shows the details of measured data from where the data can be downloaded as csv. file.

As BIMification is done as a project on the web platform, the users having the project ID and password can get access to this platform using tablets or smartphones. The BIMified model for the selected sensors is shown in Fig. 18, where the recorded data can be visualised graphically.

This platform is developed to visualise the data in AR and MR interfaces as the MR application can be directly linked to MR devices like Microsoft HoloLens and Trimble Site Vision. The idea of this future direction is already developed in which BIMified SHM system will be made accessible to the AR/MR platforms and the visualization of the SHM datasets will be carried out using cyber-physical devices. In this way, real-time monitoring of bridges can be done onsite.

## Conclusions

This research has addressed major developments in the automation of bridge Structural Health Monitoring (SHM) systems by using the applications of Building Information Modeling (BIM) technology. The example of a real-life bridge is considered which was designed by the traditional methods without the implementation of BIM technology. The proposed solutions of this study are making this research viable for the bridge industry by implementing BIM technology for user-friendly and smart interfaces of the SHM system and Finite Element Analysis (FEA).

This research analyses the existing SHM system of bridge. For this purpose, FEA and field load testing techniques are employed. The results of both static and dynamic measurements are compared with the numerical calculations and the percentage difference in results is compared with the findings of EC. Whereas the comparison of permanent and total deflections constitutes from 0 to 6%, therefore, meets the standard condition of not exceeding the level of 10%^29,56^. Such results prove that the stiffness of the spans is consistent with the values of the calculation model, thus validating the FE model of the bridge and showing the sufficiency of existing sensors measuring the static parameters of SHM system. In the case of dynamic measurements, the high modal density values and the limited number of accelerometers are limiting factors in the case of the modal identification process, thus requiring more number of dynamic measurement devices for the installed SHM system of the bridge.

The novelty of this research has achieved the automatic generation of the FE model. As FE model development is an iterative and time-consuming process, this solution will ease the process of numerical modelling. It has been done using the applications of BIM technology, where a novel Visual Programming Language (VPL) script is developed using the indirect integration method in Dynamo. This script is based on functional blocks connected in a specific order to perform desired tasks, including mathematical operations, creating, and manipulating geometries, and exchanging data between BIM environment and other types of engineering software. These building blocks can generate the geometry of any FE model enabling the integration between BIM and FEM environment. This integration results in the automation of FEM frameworks.

Further, BIMification of the bridge SHM system is developed by deploying selected sensors (strain sensor, temperature sensor, and vibration sensor) to the BIM model of the bridge. This BIM-based sensory model enables the control and monitoring of the bridge SHM system. This approach is mainly targeting IoT-based web platforms which can offer remote data recording and monitoring services. Such a platform is developed in this research using a free version of an IoT platform, having the inclusion of Arduino codes for the communication between sensors and the web platform. This platform can hold a complete SHM system as a single unit where all the sensors are embedded as individual subunits with the graphical representation of measured data.

Thus, the major aim of this research is achieved by bringing the technological advancements of the IoT domain, smart applications of BIM technology, and Common Data Environments (CDE), i.e., MR application, to automate the SHM system of bridges. Moreover, the applications of CDE help the users to visualise the recorded signal data in connection with the bridge's Asset Information Model (AIM). Implementing such systems will not only serve the purpose of real-time bridge health monitoring but also automate the bridge monitoring, data recording, data processing, and results evaluation stages which can save time and financial resources of the bridge authorities.

## Future research work

The future goal of this research work is to link BIMified SHM system with the applications of Augmented Reality (AR) and Mixed Reality (MR). It can be done by using Cyber-Physical devices like HoloLens and Site Vision. To achieve this goal, the MR application is already used in this study as it is serving as the base platform for the implementation of AR/MR. So, the authors are already conducting the experimentation of the next phase of this study in which real-time bridge monitoring data of any SHM system can be visualised using AR/MR applications.

## Data Availability

The datasets used and analysed during the current study be available from the corresponding author upon reasonable request.

## References

[1] Li H, Ou J (2016). The state of the art in structural health monitoring of cable-stayed bridges. J. Civ. Struct. Health Monit..

[2] Li H, Li S, Ou J, Li H (2012). Reliability assessment of cable-stayed bridges based on structural health monitoring techniques. Struct. Infrastruct. Eng..

[3] Panah RS, Kioumarsi M (2021). Application of building information modelling (BIM) in the health monitoring and maintenance process: A systematic review. Sensors.

[4] Li X, Xiao Y, Guo H, Zhang J (2022). A BIM based approach for structural health monitoring of bridges. KSCE J. Civ. Eng..

[5] Wang J, You H, Qi X, Yang N (2022). BIM-based structural health monitoring and early warning for heritage timber structures. Autom. Constr..

[6] Angelosanti M, Currà E, Sabato A (2023). BIM oriented applications of structural health monitoring based on magnified digital image correlation point-clouds. Autom. Constr..

[7] Honghong S, Gang Y, Haijiang L, Tian Z, Annan J (2023). Digital twin enhanced BIM to shape full life cycle digital transformation for bridge engineering. Autom. Constr..

[8] Sadhu A, Peplinski JE, Mohammadkhorasani A, Moreu F (2023). A review of data management and visualization techniques for structural health monitoring using BIM and virtual or augmented reality. J. Struct. Eng..

[9] Figueiredo E, Manuel M, Marques B, Sobre E, Warburg AM, Mendes A (2013). Condition Assessment of Bridges: Past, Present and Future. A Complementary Approach.

[10] Zhang G, Liu Y, Liu J, Lan S, Yang J (2022). Causes and statistical characteristics of bridge failures: A review. J. Traffic Transp. Eng. (English Edition).

[11] Al-Khateeb HT, Shenton HW, Chajes MJ, Aloupis C (2019). Structural health monitoring of a cable-stayed bridge using regularly conducted diagnostic load tests. Front. Built. Environ..

[12] Čápová K, Velebil L, Včelák J (2020). Laboratory and in-situ testing of integrated FBG sensors for SHM for concrete and timber structures. Sensors.

[13] Peng T, Nogal M, Casas JR, Turmo J (2021). Planning low-error SHM strategy by constrained observability method. Autom. Constr..

[14] Iannacone L, Francesco Giordano P, Gardoni P, Pina LM (2022). Quantifying the value of information from inspecting and monitoring engineering systems subject to gradual and shock deterioration. Struct. Health Monit..

[15] Bień J, Kużawa M (2020). Dynamic tests in bridge health monitoring. Studia Geotechnica et Mechanica.

[16] Bień J, Kużawa M, Kamiński T (2015). Validation of numerical models of concrete box bridges based on load test results. Arch. Civil Mech. Eng..

[17] Lantsoght EOL, van der Veen C, de Boer A, Hordijk DA (2017). State-of-the-art on load testing of concrete bridges. Eng. Struct..

[18] Innocenzi RD, Nicoletti V, Arezzo D, Carbonari S, Gara F, Dezi L (2022). A good practice for the proof testing of cable-stayed bridges. Appl. Sci..

[19] Bakht B, Pinjarkar SG. Dynamic Testing of Highway Bridges - A Review. Transp Res Rec 1989:93–100.

[20] Filar Ł, Kałuża J, Wazowski M (2017). Bridge load tests in Poland today and tomorrow—The standard and the new ways in measuring and research to ensure transport safety. Procedia Eng..

[21] De Angelis A, Pecce MR (2023). Model assessment of a bridge by load and dynamic tests. Eng. Struct..

[22] Poprawa G. Static and Dynamic Load Testing in a Lifecycle of a Bridge Infrastructure. 2020 6th International Engineering Conference “Sustainable Technology and Development" (IEC), IEEE; 2020, p. 239–239. 10.1109/IEC49899.2020.9122928

[23] Zarate Garnica GI, Lantsoght EOL, Yang Y (2022). Monitoring structural responses during load testing of reinforced concrete bridges: A review. Struct. Infrastruct. Eng..

[24] Enckell-El Jemli M, Karoumi R, Lanaro F. Monitoring of the new Årsta railway bridge using traditional and fiber optic sensors. In: Liu S-C, editor., 2003, p. 279. 10.1117/12.482707.

[25] Cai H, Abudayyeh O, Abdel-Qader I, Attanayake U, Barbera J, Almaita E (2012). Bridge deck load testing using sensors and optical survey equipment. Adv. Civil Eng..

[26] Kuryłowicz-Cudowska A, Miśkiewicz M, Meronk B, Pyrzowski Ł, Wilde K. Reference FEM model for SHM system of cable-stayed bridge in Rzeszów. 3rd Polish Congress of Mechanics (PCM) / 21st International Conference on Computer Methods in Mechanics (CMM), 2016.

[27] Duvnjak I, Bartolac M, Nilimaa J, Sas G, Blanksvärd T, Täljste B (2018). Lessons learnt from full-scale tests of bridges in Croatia and Sweden. J. IABSE Symp. Nantes.

[28] Dong C, Bas S, Debees M, Alver N, Catbas FN (2020). Bridge load testing for identifying live load distribution, load rating, serviceability and dynamic response. Front. Built. Environ..

[29] Nguyen DC, Salamak M, Katunin A, Gerges M (2022). Finite element model updating of rc bridge structure with static load testing: A case study of vietnamese ThiThac bridge in coastal and marine environment. Sensors.

[30] Ghannad P, Lee Y-C, Dimyadi J, Solihin W (2019). Automated BIM data validation integrating open-standard schema with visual programming language. Adv. Eng. Inform..

[31] Hasan AMM, Torky AA, Rashed YF (2019). Geometrically accurate structural analysis models in BIM-centered software. Autom. Constr..

[32] Korus K, Salamak M, Jasiński M (2021). Optimization of geometric parameters of arch bridges using visual programming FEM components and genetic algorithm. Eng. Struct..

[33] Latas C, Soust-Verdaguer B, Hollberg A, Palumbo E, Quiñones R (2022). BIM-based LCSA application in early design stages using IFC. Autom. Constr..

[34] Fawad M, Koris K, Salamak M, Gerges M, Bednarski L, Sienko R (2022). Nonlinear modelling of a bridge: A case study-based damage evaluation and proposal of Structural Health Monitoring (SHM) system. Arch. Civil Eng..

[35] Simon A, Courtois A, Clauzon T, Coustabeau E, Vinit S. Long-term measurement of strain in concrete: durability and accuracy of embedded vibrating wire strain gauges. SMAR 2015 - Third Conference on Smart Monitoring, Assessment and Rehabilitation of Civil Structures, Antalya: 2015.

[36] Ye X, Sun Z, Cai X, Mei L (2019). An improved step-type liquid level sensing system for bridge structural dynamic deflection monitoring. Sensors.

[37] Kamiński W (2021). The concept of accuracy analysis of the vertical displacements gained from the hydrostatic levelling systems’ measurements. Sensors.

[38] Janssens ML, Apte V (2022). Fundamental measurement techniques. Flammability Testing of Materials Used in Construction, Transport and Mining.

[39] Wierzbicki S, Pióro Z, Osiniak M, Antoszkiewicz E (2020). Inclinometer method of displacement measurements as an alternative to optical measurements in structural health monitoring—On site tests. Arch. Civil Eng..

[40] Bedon C, Bergamo E, Izzi M, Noè S (2018). Prototyping and validation of MEMS accelerometers for structural health monitoring—The case study of the Pietratagliata cable-stayed bridge. J. Sensor Actuat. Netw..

[41] Naraharisetty V, Talari VS, Neridu S, Kalapatapu P, Pasupuleti VDK. Cloud Architecture for IOT Based Bridge Monitoring Applications. 2021 International Conference on Emerging Techniques in Computational Intelligence (ICETCI), IEEE; 2021, p. 39–42. 10.1109/ICETCI51973.2021.9574044.

[42] Mahmud MA, Bates K, Wood T, Abdelgawad A, Yelamarthi K. A complete Internet of Things (IoT) platform for Structural Health Monitoring (SHM). 2018 IEEE 4th World Forum on Internet of Things (WF-IoT), IEEE; 2018, p. 275–9. 10.1109/WF-IoT.2018.8355094.

[43] Vipond N, Kumar A, James J, Paige F, Sarlo R, Xie Z (2023). Real-time processing and visualization for smart infrastructure data. Autom. Constr..

[44] Scherer RJ, Katranuschkov P (2018). BIMification: How to create and use BIM for retrofitting. Adv. Eng. Inform..

[45] Weihnacht B, Tschöke K, Meyendorf N, Ida N, Singh R, Vrana J (2022). Smart monitoring and SHM. Handbook of Nondestructive Evaluation 40.

[46] Kensek K (2020). A BIM-based visualization tool for facilities management: Fault detection through integrating real-time sensor data into BIM. J. Archit. Eng. Technol..

[47] Martín C, Garrido D, Llopis L, Rubio B, Díaz M (2022). Facilitating the monitoring and management of structural health in civil infrastructures with an Edge/Fog/Cloud architecture. Comput. Stand. Interfaces.

[48] Deng L, Lai S, Ma J, Lei L, Zhong M, Liao L (2022). Visualization and monitoring information management of bridge structure health and safety early warning based on BIM. J. Asian Archit. Build. Eng..

[49] Smarsly K, Hartmann D, Law KH (2013). An integrated monitoring system for life-cycle management of wind turbines. Smart Struct. Syst..

[50] Singh P, Sadhu A (2020). System identification-enhanced visualization tool for infrastructure monitoring and maintenance. Front. Built. Environ..

[51] Luleci F, Li L, Chi J, Reiners D, Cruz-Neira C, Catbas FN (2022). Structural health monitoring of a foot bridge in virtual reality environment. Proc. Struct. Integr..

[52] Awadallah O, Sadhu A (2023). Automated multiclass structural damage detection and quantification using augmented reality. J. Infrastruct. Intell. Resil..

[53] Catbas FN, Luleci F, Zakaria M, Bagci U, LaViola JJ, Cruz-Neira C (2022). Extended reality (XR) for condition assessment of civil engineering structures: A literature review. Sensors.

[54] EN 1991–2: Eurocode 1: Actions on structures - Part 2: Traffic loads on bridges. 2003.

[55] Polish Committee for Standardization M and QC. PN-85/S-10030. Obiekty mostowe. Obciążenia (Polish Standard: Bridge Structures. Loads). 1985.

[56] EN 1992–1–1: Eurocode 2: Design of concrete structures - Part 1–1: General rules and rules for buildings. 2004.

[57] EN 1992–2: EC-2: Design of concrete structures - Part 2: Concrete bridges - Design and detailing rules. 2005.

