# Author Correction: Automation of structural health monitoring (SHM) system of a bridge using BIMification approach and BIM-based finite element model development

**DOI:** 10.1038/s41598-023-44858-1

**Published:** 2023-10-17

**Authors:** Muhammad Fawad, Marek Salamak, Grzegorz Poprawa, Kalman Koris, Marcin Jasinski, Piotr Lazinski, Dawid Piotrowski, Muhammad Hasnain, Michael Gerges

**Affiliations:** 1 Faculty of Civil Engineering, Silesian University of Technology, Ul. Akademicka 2A, 44-100 Gliwice, Poland; 2 Faculty of Civil Engineering, Budapest University of Technology and Economics, Műegyetem Rkp. 3, 1111 Budapest, Hungary; 3 University of Florida, Gainesville, FL 32611 USA; 4 University of Wolverhampton, Wulfruna St, Wolverhampton, WV1 1LY UK

Correction to: *Scientific Reports* 10.1038/s41598-023-40355-7, published online 14 August 2023

The Funding section in the original version of this Article was omitted.

The Funding section now reads: “This publication was supported under the Initiative of Excellence—Research University program implemented at the Silesian University of Technology, Poland under project No. 32/014/SDU/10-22-58”.

The original Article has been corrected.

